# The initial effect of U.S. tax reform on foreign acquisitions

**DOI:** 10.1007/s11142-023-09760-1

**Published:** 2023-04-17

**Authors:** Harald J. Amberger, Leslie Robinson

**Affiliations:** 1 Vienna University of Economics and Business, Welthandelsplatz 1, 1020 Vienna, Austria; 2 Tuck School of Business at Dartmouth College, 100 Tuck Hall, Hanover, NH 03755 USA

**Keywords:** Tax Cuts and Jobs Act, Mergers and acquisitions, Investment, Repatriation Tax, GILTI, FDII, G34, H25, H32, K34, M21, M41

## Abstract

The Tax Cuts and Jobs Act (TCJA) of 2017 marked a significant change in U.S. domestic and international tax policy, altering incentives for U.S. firms to own foreign assets. We examine the initial response of U.S. firms’ foreign acquisition patterns to the TCJA’s key reform provisions. We find a significant overall decrease in the probability that a foreign target is acquired by a U.S. firm after the reform, suggesting that the net effect of the TCJA was to reduce acquisitions abroad. Cross-sectional variation across target and acquirer characteristics points to the elimination of the repatriation tax and the TCJA’s global intangible low-taxed income (GILTI) regime as critically influencing cross-border acquisitions by U.S. firms. Specifically, U.S. acquirers with little foreign presence prior to the TCJA are more likely to acquire a foreign target, while U.S. acquirers are less likely to acquire profitable targets in low-tax countries. Results from our empirical analyses are consistent with the TCJA prompting fewer but more value-enhancing, less tax-motivated foreign acquisitions by U.S. firms.

## Introduction

For the two decades preceding the 2017 Tax Cuts and Jobs Act (TCJA), the United States debated whether and how to reform its international tax system. The country then had a worldwide system, which often resulted in U.S. taxes being due when foreign income was repatriated, thus discouraging U.S. multinational corporations from doing so and, in some cases, encouraging suboptimal investments abroad. This tax system was thought to put U.S. firms at a disadvantage, compared to firms based in countries with territorial systems that impose no domestic tax on foreign profits.^1^ Signed into law on December 22, 2017, the TCJA introduced some features of a territorial system, that is, elimination of U.S. taxation of future income earned outside the United States (the repatriation tax), along with new features of a worldwide system, that is, the global intangible low-taxed income (GILTI) regime, which taxes some foreign income in the United States as it is earned if foreign tax rates are below a minimum rate (115th Congress 2017).^2^ These changes to the U.S. international tax system were complemented by a substantial U.S. statutory corporate income tax rate reduction from 35 to 21%. Understanding firms’ investment incentives under this new hybrid system is imperative in light of ongoing debates about U.S. tax policy (Alston and Bird 2021).

We study the TCJA’s initial effect on U.S. firms’ decisions to acquire foreign targets. Observing changes in foreign mergers and acquisitions (M&A) is particularly enlightening because some prominent cross-border M&A patterns were an oft-cited indicator that the U.S. international tax system was flawed. Several empirical studies in the years immediately preceding the TCJA document these patterns. Lyon (2020), for example, highlights that an increasing share of cross-border M&As transferred assets and ownership of U.S. firms to foreign ownership. Bird et al. (2017) document that U.S. firms were disproportionately targets for acquisition by foreign firms, while Feld et al. (2016) highlight that U.S. firms were disadvantaged in bidding for foreign targets. Hanlon et al. (2015) find that U.S. firms looking to avoid the U.S. repatriation tax were more likely to pursue value-destroying foreign expansion through acquisitions. Therefore a change in cross-border M&A patterns may indicate that the TCJA addressed these flaws and reduced tax frictions more generally surrounding foreign investment by U.S. firms, an aim of the tax reforms. We conclude from our study that the TCJA did indeed lessen some of these frictions, making the U.S. tax system more neutral with respect to U.S. firms’ foreign acquisitions.

The TCJA allows us to empirically investigate the effect of changes in tax law on outbound acquisitions, because passage of the act was a relatively exogenous event (Carrizosa et al. 2022; Wagner et al. 2018). Although tax reform was likely after the 2016 U.S. election, the framework was not presented until September 2017; the act itself was signed into law on December 22, 2017 (115th Congress 2017; Gaertner et al. 2020), limiting the opportunity for anticipatory actions. In addition, the TCJA did not cause immediate unilateral policy responses, as the major U.S. trading partners did not substantially change their tax rules (Chalk et al. 2018). Changes to other countries’ tax systems thus do not cloud the ability to detect the TCJA’s economic effects, strengthening the inferences we can draw by examining the reform. One drawback of the setting is that the TCJA contains multiple important policy changes. As a result, it can be challenging to identify specific reform provisions as the drivers of changing foreign acquisition patterns.

In general, the TCJA could have changed the incentives for foreign acquisitions in several ways, making the overall effect of the reform on foreign M&A activity an empirical question. On the one hand, repealing the U.S. repatriation tax reduces the expected tax rate on future income earned abroad, thereby lowering the marginal cost of foreign investment (Liu 2020). This change strengthens the incentive for foreign acquisitions, because U.S. firms are no longer tax-disadvantaged owners of foreign targets (Desai and Hines 2003; Feld et al. 2016). On the other hand, repealing the repatriation tax removes an internal capital market friction. By eliminating the tax cost of repatriating foreign earnings, the TCJA raises the opportunity cost of reinvesting these profits abroad (Albertus et al. 2022; Arena and Kutner 2015; Edwards et al. 2016), weakening the incentive for foreign acquisitions. The GILTI regime discourages U.S. firms from acquiring profitable targets in low-tax jurisdictions because it serves as a minimum tax that only applies if the target’s income accrues to a U.S. owner. Lowering the statutory corporate income tax rate from 35 to 21% provides firms with more after-tax cash flow in the United States (Dyreng et al. 2020), which can be used to finance acquisitions abroad. In sum, the TCJA provides us with multiple sources of variation across acquirer and target characteristics.

To study the effect of the TCJA on outbound M&A, we collect data on cross-border acquisitions completed between 2011 and 2019 from Bureau van Dijk’s Zephyr database. Our global sample includes 3,266 targets, located in 46 countries. Moreover, to investigate whether foreign investment responses vary across different types of *potential* U.S. acquirers and identify reform provisions that might explain any variation, we combine our dataset on cross-border deals with financial statement data from Compustat and obtain a sample of potential U.S. acquirers. We begin our analysis by examining whether the reform changed the likelihood that a foreign target is acquired by a U.S. firm.^3^ Our analysis provides strong evidence that the overall probability of being acquired by a U.S. firm decreased by 3.5–4.5 percentage points post reform. This result, which holds across multiple specifications and robustness tests, indicates that the TCJA generally weakened the incentives of U.S. firms to pursue foreign M&A, while we find no change in the foreign M&A of non-U.S. firms.

To better understand this result, we document both target and acquirer characteristics associated with differential responses to the TCJA’s key provisions. The TCJA’s most significant international provision was the repeal of the U.S. repatriation tax on future income earned abroad. Relatedly, we find a lower post-reform likelihood that U.S. firms with untaxed foreign earnings acquire a foreign target, a higher likelihood that U.S. firms with no international presence acquire a foreign target, and a decreased probability that U.S. firms acquire low-growth foreign targets.^4^ We triangulate these results by examining deal announcement returns (Hanlon et al. 2015) and find evidence that, for acquirers with large untaxed foreign earnings, returns are relatively higher after the TCJA. Thus we conclude that, while the repeal of the repatriation tax both increased and decreased incentives for foreign M&A, firms with high amounts of locked-out cash prior to the TCJA tend to pursue more value-enhancing acquisitions after the reform.

We design several additional tests to examine other key provisions of the TCJA. Most notably, we find that the GILTI provision reduces incentives for U.S. firms to acquire profitable low-taxed foreign targets.^5^ Our empirical setup also allows us to evaluate foreign-derived intangible income (FDII, described in more detail in Section 2.2 and Appendix 1), a provision intended to work in tandem with GILTI to neutralize tax as a driver of where to generate sizeable profits. Here we find profitable *U.S. targets* serving foreign markets being more likely to be acquired by U.S. firms after the TCJA than other U.S. targets. Finally, we document that the reduction in the statutory corporate income tax rate incentivizes debt-constrained U.S. acquirers to expand abroad. Taken together, we conclude that firms are responding as intended to the policy objectives of the TCJA, by increasing foreign acquisitions in some cases while decreasing them in other cases as well as changing the characteristics of targets acquired.

Our study contributes to the literature by assessing the initial effect of the 2017 U.S. tax reform on foreign acquisitions by U.S. firms. Our results suggest that the TCJA influenced foreign investment by lowering the average propensity of U.S. firms to acquire foreign targets while leaving the M&A of non-U.S. firms unchanged. The introduction of a hybrid tax system led to heterogeneous investment responses that have important tax policy implications. The key territorial feature of the TCJA—the elimination of the U.S. repatriation tax—appears to have removed tax distortions for U.S. firms in the global M&A market. Multinationals with untaxed foreign earnings make fewer but more value-enhancing foreign acquisitions after the TCJA, while those with limited foreign operations or that faced debt constraints are more likely to acquire foreign targets. In contrast, GILTI—the unique worldwide feature of the TCJA—resulted in a tax disadvantage for U.S. firms when bidding for profitable low-taxed foreign targets. This relative disadvantage would be eliminated as other countries uniformly adopt the OECD’s global minimum tax, which is like the GILTI in many respects, putting potential acquirers of such targets in the global M&A market on an equal footing.

## Related literature and hypothesis development

### Taxes and cross-border M&A

Studies that analyze cross-border M&A often control for differences in taxation but pay little attention to the role of taxation itself. Bertrand et al. (2007) include taxes among their explanatory variables, for example, when estimating a conditional logit model over 400 European cross-border acquisitions in the 1990s. Other studies choose to focus on a single aspect of taxation, such as taxes imposed on buyers and sellers at the time of the deal or taxes on the subsequent profits of the combined entity. For instance, some studies focus on the *corporate* capital gains tax (Erickson 1998; Erickson and Wang 2000; Maydew et al. 1999; Todtenhaupt et al. 2020), while others focus on the *personal* capital gains tax (Ayers et al. 2003, 2004, 2007). Collectively, this work documents that taxes on the selling shareholders affect the probability a deal will occur as well as the structure of the deal and the acquisition premium; all but Todtenhaupt et al. (2020) focus on domestic U.S. acquisitions.

Another distinction is whether a study addresses the target’s tax system or that faced by the acquiring firm. Here the cross-border nature of M&A matters. The statutory tax rate in the target’s country is most often explored (Arulampalam et al. 2019; Coeurdacier 2009; di Giovanni 2005; Erel et al. 2012; Herger et al. 2016). This literature generally finds a negative elasticity of M&A with respect to the target country’s tax system. Bradley et al. (2021), for example, find that the introduction of a patent box in the target country increases the likelihood of targets being acquired, if no additional nexus requirements are imposed. Huizinga et al. (2012) find that nonresident dividend withholding taxes imposed by a target country damp cross-border M&A.

Studies that focus on the tax system faced by the acquiring firm relate most closely to ours. In the economics literature, the ownership neutrality concept introduced by Desai and Hines (2003) describes a tax system that does not distort the ownership of assets. Capital ownership neutrality requires a level playing field for all bidders pursuing a foreign acquisition. When the acquirer is located in a country with a worldwide tax system, a cross-border acquisition can trigger additional taxation of the target’s income in the country of the acquirer (Huizinga et al. 2012; Huizinga and Voget 2009; Voget 2011). For foreign acquisitions financed through domestic funds, a repatriation tax imposes an additional tax cost on future income earned by the target (Liu 2020). These taxes handicap the acquisition of foreign targets by acquirers expecting to face these repatriation tax burdens.

Only three major acquiring countries in the global M&A market have imposed potentially significant repatriation taxes on a foreign target’s income: the United Kingdom, Japan, and the United States. Feld et al. (2016) found that the repeal of the repatriation tax in Japan and the United Kingdom increased the number of foreign acquisitions, with a much larger effect in Japan. When these authors simulated a similar policy switch in the United States, the number of cross-border acquisitions increased by 11%.

Two aspects make the U.S. tax system and U.S. firms quite different from those in Japan and the United Kingdom. First, in the 2017 reform of its tax code, the U.S. did not abolish its worldwide tax system. Instead it moved to a quasi-territorial system, due to the GILTI regime (described below). As the GILTI regime significantly departed from U.S. international tax policy, concurrent work examines the changing incentives surrounding foreign acquisitions with respect to it (Dunker et al. 2022; Atwood et al. 2020). These studies identify public U.S. acquirers more likely to be affected by the GILTI provision and search for a change in the likelihood of a foreign acquisition. Dunker et al. (2022) find a negative effect on foreign acquisitions, while Atwood et al. (2020) find a positive effect. In contrast to our work, both studies rely on consolidated data of U.S. public acquirers and cannot directly link the effects of the GILTI provision to specific foreign targets or their characteristics.^6^

The second unique aspect of U.S. firms is that some were already quite active in acquiring foreign targets prior to the reform.^7^ Hanlon et al. (2015), Edwards et al. (2016), and Harford et al. (2017) show that U.S. firms with a greater accumulation of foreign cash, due to repatriation tax avoidance (locked-out earnings or locked-out cash), are more likely to acquire foreign targets. However, all three studies find these investments to be less value-enhancing in terms of deal announcement returns, buy and hold returns, and returns on assets. Bird et al. (2017) test a related hypothesis in the U.S. domestic M&A market. They find that U.S. firms with greater locked-out earnings are more likely to be acquired by foreign firms located in countries with a territorial tax system than they are by U.S. firms, because the foreign firms can avoid the repatriation tax on their U.S. targets’ foreign profits. These researchers corroborate their results by examining countries that switched from a worldwide to a territorial tax system (i.e., the United Kingdom and Japan). After the switch, acquirers from switching countries increase their preference for U.S. targets with significant locked-out earnings.

### Pertinent TCJA provisions and hypothesized effects on outbound M&A

**Fig. 1:**
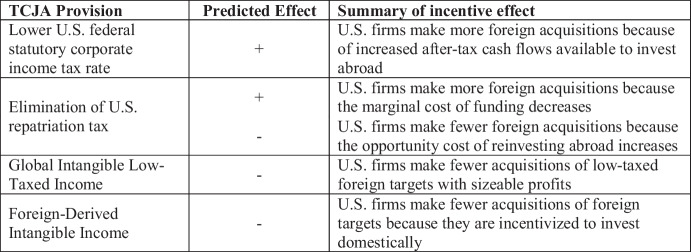
TCJA provisions and the incentives for U.S. firms to engage in outbound M&A. This figure summarizes the hypothesized incentive effects of the individual TCJA provisions for the outbound M&A of U.S. firms, as discussed in Section 2.2

The core provisions of the TCJA were meant to address the investment distortions caused by the U.S. corporate tax system, including its perceived role in so-called inversion transactions, which had attracted significant public scrutiny in the years prior to the reform.^8^ In this section and in Fig. 1, we provide an overview of the provisions we expect to change the incentives for foreign acquisitions by U.S. firms. We discuss each TCJA provision in detail in Appendix 1.

The most significant domestic reform was the reduction in the U.S. federal statutory corporate income tax rate from 35 to 21%. The resulting tax rate puts the United States in line with the average statutory corporate income tax rate in the OECD, reducing firms’ incentives to shift operations (and income) out of the country. The most significant international reform was the abolishment of the U.S. repatriation tax on post-TCJA foreign earnings. As part of the transition to a new system of taxing foreign earnings, the United States imposed a one-time transition tax on the untaxed foreign earnings U.S. multinationals had accumulated pre reform. Prior to the TCJA, a U.S. parent company faced a 35% U.S. corporate income tax (minus applicable foreign tax credits) on the dividends received from its foreign subsidiaries. To avoid this tax, U.S. multinationals often retained their earnings in low-tax countries—referred to as the lock-out effect. By no longer subjecting future foreign income of U.S. multinationals to U.S. taxation, the TCJA was heralded as incorporating territoriality into the U.S. tax system, similar to the practices followed by other developed countries.

The reduction in the statutory corporate income tax rate impacted all existing and potential U.S. operations by increasing firms’ expected after-tax cash flows (Dyreng et al. 2020). To the extent that the increased cash flow attenuated financial constraints and provided additional liquidity that could be used to acquire foreign targets, the reduction in the tax rate would have increased *outbound* M&A. How the repeal of the repatriation tax would affect the incentives for foreign acquisitions is less clear. On the one hand, eliminating the lock-out effect increased the opportunity cost of investing abroad, weakening the incentives for foreign acquisitions. On the other, repealing the repatriation tax on future foreign income reduced the marginal cost of funding foreign acquisitions through domestic funds, increasing the incentives for foreign acquisitions (Liu 2020). Hence, depending on a firm’s investment opportunities and the marginal source of funding for foreign investment, eliminating the repatriation tax could result in either an increase or decrease in cross-border M&A.

The TCJA also included two provisions targeted at encouraging one type of investment while discouraging another. Acting as the carrot and the stick, respectively, the FDII regime and the GILTI regime should indirectly encourage U.S. firms to own intellectual property in the U.S. rather than outside the U.S. in a low-tax jurisdiction. GILTI and FDII target intellectual property migration indirectly because income earned from this property is difficult to observe and therefore to target with tax policy. Thus both tax regimes target income exceeding a 10% return on tangible assets. The rules result in a tax penalty under GILTI for earning excess foreign income taxed at a low rate and a tax subsidy under FDII for earning excess income in the United States.^9^

Specifically, GILTI imposes an immediate U.S. tax on excess income earned outside the United States, if the income is not subject to a sufficient level of taxation in the foreign jurisdiction (currently around 13%).^10^ The provision should discourage outbound acquisitions by U.S. firms of profitable targets in low-tax jurisdictions. This disincentive arises because the excess income and the tax rate are determined as an average across a U.S. multinational’s aggregate foreign operations, potentially increasing the effective tax cost of earnings generated by low-taxed targets.^11^ FDII, in contrast, imposes a tax rate lower than 21% on excess income earned in the United States from export sales of goods and services. The provision should incentivize U.S. firms to operate domestically and serve foreign markets through U.S. operations. Thus FDII might reduce the incentives for outbound acquisitions and attract investment back to the United States.

Each of these provisions—reduction in the U.S. statutory corporate income tax rate, elimination of the U.S. repatriation tax, and introduction of FDII and GILTI—changes the incentives for U.S. firms to acquire abroad. The lower statutory corporate income tax rate should facilitate foreign acquisitions by increasing the cash available to invest abroad. Elimination of the repatriation tax could either increase or decrease outbound M&A by U.S. firms, depending on the marginal source of funds and access to capital as well as foreign investment opportunities. FDII should decrease outbound M&A, by making the relative cost of operating in the United States versus abroad more favorable to the former, while GILTI should reduce U.S. acquisitions of profitable targets in low-tax countries. However, the net effect of these incentives is an empirical question and will depend on how firm- and target-specific facts interact with the TCJA provisions. Therefore our empirical tests consider how these changes to the U.S. tax system alter the incentives for foreign acquisitions, conditional on the characteristics of foreign targets and potential U.S. acquirers.

## Empirical setup, data, and descriptive statistics

### Empirical setup

Our empirical strategy is twofold. First, we examine foreign targets and assess whether their likelihood of being acquired by a U.S. firm changed in response to the TCJA. This analysis illuminates how the reform affected U.S. firms’ level of activity in foreign M&A markets. We also can assess whether the reform changed the incentives to acquire certain types of foreign targets. We focus on deal probabilities, because the expected impact of any tax reform on deal valuations is a priori less clear. For instance, transaction volume could decrease alongside increasing valuation if larger (higher value) acquisitions benefit more from the reform than do smaller (lower value) ones; that is, if the expected tax benefits of larger deals are significantly greater, on average, than those of smaller deals.

Second, we analyze U.S. firms and test whether the reform changed the incentives for foreign acquisitions, conditional on the characteristics of a potential U.S. acquirer. In this acquirer-level analysis, we test whether foreign investment responses, documented at the target level, vary across different types of potential U.S. acquirers and investigate which reform provisions might drive these responses. In sum, our twofold empirical strategy allows us to consider some of the nuances of the TCJA, as outlined in Fig. 1.

#### Target-level analysis

To test for the effect of the TCJA on U.S. acquisitions of foreign targets, we examine the likelihood that foreign target *i* is acquired by a U.S. firm. To this end, we estimate the following linear probability model^12^:

1 $$\begin{aligned}{US\_ACQ}_{i}= {\alpha }_{j}+{\alpha }_{c}+&{{\beta }_{1}POST}_{t}+ {\beta }_{2}{LN\left(ASSETS\right)}_{i,t-1}+{\beta }_{3}{ROA}_{i,t-1} +\\ &{\beta }_{4}{LEVERAGE}_{i,t-1}+{\beta }_{5}{INTANGIBLES}_{i,t-1}+{\beta }_{6}{LOSS}_{i,t-1}+{\varepsilon }_{i}.\end{aligned}$$

*US_ACQ* is an indicator variable equal to one if foreign target *i* has a U.S. acquirer and zero if the target is acquired by a non-U.S. firm. Following Hanlon et al. (2015), we define *US_ACQ* based on the country of incorporation of the acquirer’s global ultimate owner (i.e., parent company). Hence an acquisition by a foreign subsidiary of a U.S. firm is classified as a U.S. acquisition, considering that firms could use cash held in their foreign subsidiaries to acquire foreign targets.

Our independent variable of interest, *POST*, is an indicator variable equal to one if target *i* is acquired after the TCJA and zero otherwise. ${\beta }_{1}$ captures the effect of the TCJA on the probability that foreign target *i* has a U.S. acquirer. A negative (positive) coefficient on ${\beta }_{1}$ suggests that the tax reform reduced (increased) the probability of being acquired by a U.S. firm.

We include target-industry fixed effects (${\alpha }_{j}$), defined at the one-digit NACE industry level, and target-country fixed effects (${\alpha }_{c}$). These fixed effects absorb the impact of time-invariant target-industry and target-country characteristics. By including these fixed effects, we identify the effect of the TCJA from over-time variation in the probability that a foreign target is acquired by a U.S. firm within each target industry and country. In a robustness test, we replace the separate fixed effects with target-country-industry fixed effects and find consistent results (see column (5) of Table 2 panel A).

In addition to these industry- and country-level controls, we follow Bird et al. (2017) and control for characteristics of the target that could influence its likelihood of having a U.S. acquirer. Specifically, we control for target size by including the natural logarithm of total assets (*LN(ASSETS)*). We also control for profitability (*ROA*), noncurrent liabilities (*LEVERAGE*), and intangible assets (*INTANGIBLES*), all scaled by total assets. These variables capture differences in profit shifting strategies between U.S. and non-U.S. acquirers (Kohlhase and Pierk 2020; Markle 2016) that could affect the relative attractiveness of a foreign target. Finally, we add *LOSS* as an indicator variable equal to one if target *i* reports a loss. Losses may alter the future effective tax rate of the target and thus affect its attractiveness (Bird 2015). Aside from these tax aspects, most of our control variables proxy also for (future) target performance (Bird et al. 2017). We lag control variables by one year to capture target characteristics in the year prior to the deal. We define the variables and outline the respective data sources in Appendix 2.^13^

#### Acquirer-level analysis

To analyze the effect of the TCJA on outbound acquisitions of potential U.S. acquirers, we estimate the following linear probability model, which models the likelihood that U.S. firm *i* acquires a foreign target in year *t*:

2 $$\begin{aligned}{FOR\_ACQ}_{i,t}= {\alpha }_{i}+{\alpha }_{t}+&{{\beta }_{1}POST}_{t}+ {{\beta }_{2}\sum TREATED}_{i}+{{\beta }_{3}{POST}_{t}*\sum TREATED}_{i}+\\ &{\beta }_{4}{SALES\_GROWTH}_{i,t-1}+{\beta }_{5}{WORKING\_CAPITAL}_{i,t-1}+\\ &{\beta }_{6}{LEVERAGE}_{i,t-1}+{\beta }_{7}{MTB}_{i,t-1}+{\beta }_{8}{SIZE}_{i,t-1}+\\ &{\beta }_{9}{NOL}_{i,t-1}+{\varepsilon }_{i,t}.\end{aligned}$$

The dependent variable, *FOR_ACQ*, is an indicator variable equal to one if U.S. firm *i* acquires at least one foreign target in year *t* and zero otherwise. *POST* is equal to one for years after the TCJA and zero for years prior to the reform. Vector *TREATED* includes a set of treatment indicators (*REPAT_TAX_COST*, *DOMESTIC*, and *NON_INVGRADE_RATING*) to identify differential responses in M&A to the reform, conditional on pre-reform characteristics of firm *i*.^14^*REPAT_TAX_COST* is equal to one if firm *i* has untaxed (by the United States) foreign earnings prior to the TCJA (treated firms), and zero otherwise (control firms).^15^*DOMESTIC* is equal to one if firm *i* is classified as domestic prior to the TCJA and zero otherwise. We classify a firm as domestic if its foreign pre-tax income is zero or missing. *NON_INVGRADE_RATING* is equal to one if firm *i* has no or a non-investment grade credit rating prior to the reform and zero otherwise.^16^

We separately interact all treatment indicators with *POST*. We expect a negative coefficient on ${\beta }_{3}$ for *REPAT_TAX_COST*, since the repeal of the repatriation tax increases the opportunity cost of reinvesting profits abroad. Specifically, this provision reduces the tax cost of distributing foreign funds to the U.S. parent, making the repatriation of foreign profits relatively more attractive and weakening the incentives of firms with untaxed foreign earnings to reinvest these earnings through foreign M&A. Conversely, we predict positive coefficients on ${\beta }_{3}$ for *DOMESTIC* and *NON_INVGRADE_RATING*. Domestic firms are more likely to finance their foreign acquisitions through domestic funds, in which case the repeal of the repatriation tax reduces the marginal cost of investing abroad. Moreover, the lower statutory corporate income tax rate should provide these firms with greater after-tax cash flows. Similarly, investment of firms with constrained access to debt markets (*NON_INVGRADE_RATING*) is particularly sensitive to internal cash flow (Faulkender and Petersen 2012; Fazzari et al. 1988). Thus cash-tax savings should provide these firms with more internal cash, facilitating outbound M&A.

We include firm fixed effects (${\alpha }_{i}$) and year fixed effects (${\alpha }_{t}$) in all tests. Firm fixed effects control for the effect of unobserved time-invariant firm characteristics on firm *i*’s likelihood of acquiring a foreign target in year *t*. Year fixed effects absorb the impact of time-specific shocks and of the business cycle on foreign M&A. With this research design, we test how the probability to acquire a foreign target changed due to the reform within treated firms, relative to control firms. As a result, we identify the effect of the TCJA from within-firm variation in the incentives for foreign acquisitions. Note that firm and year fixed effects absorb the coefficients on *POST* and *TREATED.*

In line with prior research (Hanlon et al. 2015; Harford 1999), we control for several determinants of foreign M&A. Specifically, we include annual sales growth (*SALES_GROWTH*), noncash working capital (*WORKING_CAPITAL*), and long-term debt (*LEVERAGE*). *WORKING_CAPITAL* and *LEVERAGE* are both scaled by total assets. We add the market-to-book value of equity (*MTB*) to capture differences in firm-level growth opportunities, the natural logarithm of total assets (*SIZE*) to control for firm size, and an indicator variable for whether firm *i* reports a tax loss carryforward (*NOL*) to control for accumulated losses. In line with Eq. (1), we lag control variables by one year to capture firm characteristics in the year prior to foreign acquisitions. We define variables and outline respective data sources in Appendix 2.

### Sample selection and descriptive statistics

#### Global sample of foreign targets

We construct a global sample of acquisitions using Bureau van Dijk’s Zephyr database. This database provides deal-level data on domestic and cross-border M&A, including information on the seller, the acquirer, and the target, for both publicly listed and private targets (Bradley et al. 2021; Feld et al. 2016). We construct our global sample in a way that allows us to test whether the TCJA influenced a foreign target’s probability of being acquired by a U.S. firm and to search for cross-sectional variation in this effect, based on the target’s characteristics.

In Zephyr, we first identify acquisitions completed between 2010 and 2019 that have nonmissing deal values.^17^ Since we collect a global sample, we do not restrict deals by location. We choose 2010 as a starting point to mitigate the impact of the global financial crisis. We stop in 2019 because the COVID-19 pandemic and its economic repercussions may severely distort cross-border M&A from 2020 onward. Our final sample covers acquisitions completed between 2011 and 2019, because we lag target-level controls by one year in the analysis. We focus on deals in which the acquirer ends up with a majority stake in target *i* (Bird et al. 2017). In addition, both the target and the acquirer must be classified as corporations, and both parties must have nonmissing country and industry information.

We next link all the targets and acquirers in this sample to the Orbis database, using the identifiers provided by Bureau van Dijk. From Orbis, we extract financial statement data for each target together with ownership and location data for each acquirer; the latter enables us to identify the global ultimate owner of the acquirer and to determine its country of incorporation. With this information we can identify, for instance, acquisitions by foreign subsidiaries of U.S. firms and correctly classify these transactions as U.S. firms’ outbound M&A.

This process yields an initial sample of 33,401 acquisitions with information on the acquirer’s global ultimate owner.^18^ We delete targets with implausible financial statement data (such as negative sales, negative employees, negative fixed assets, or negative total assets) and transactions with deal values of less than €100,000.^19^ Since we are interested in the impact of the tax reform on U.S. firms’ *outbound* M&A, we exclude all deals with a U.S. target. We relax this rule in our additional tests. We also drop acquisitions with insufficient data to compute our control variables. Finally, to restrict our sample to target countries with an active M&A market, we drop observations from target countries where fewer than 15 deals were completed during our sample period.

Our final global sample covers 3,266 cross-border deals (i.e., for which the target and the acquirer are in different countries). In addition, we obtain data on 4,909 domestic deals (i.e., for which the target and the acquirer are in the same country), which we include in a robustness test. All deals involve non-U.S. targets. Table 1 shows the distribution of cross-border deals by target country with, not surprisingly, the larger, more developed countries serving as the primary target hosts (panel A). Most targets are profitable, with a mean (median) return on assets of 2.5% (4.7%), low leverage, and a low level of capitalized intangibles held on the balance sheet (panel B).

**Table 1:** Descriptive statistics

**Panel A: Sample composition by target country (Global Sample)**						
Country	# of Cross-border Deals		Country		# of Cross-border Deals	
Australia	148		Lithuania		19	
Austria	23		Malaysia		68	
Belgium	119		Netherlands		58	
Bosnia	9		New Zealand		27	
Brazil	24		Norway		98	
Bulgaria	19		Philippines		6	
Canada	124		Poland		115	
Cayman Islands	57		Portugal		40	
China	58		Romania		46	
Colombia	29		Russia		98	
Croatia	14		Serbia		43	
Czech Republic	48		Slovak Republic		11	
Denmark	52		Slovenia		17	
Finland	60		South Korea		37	
France	198		Spain		235	
Germany	207		Sri Lanka		3	
Greece	26		Sweden		124	
Hungary	11		Taiwan		9	
India	90		Thailand		23	
Ireland	46		Turkey		13	
Italy	233		Ukraine		42	
Japan	24		United Kingdom		470	
Kazakhstan	9		Vietnam		17	
Latvia	19		**Total**		**3,266**	
**Panel B: Target-level descriptive statistics (Global Sample)**						
Variables	N	Mean	SD	Q1	Median	Q4
* US_ACQ*	3,266	0.196	0.397	0.000	0.000	0.000
* POST*	3,266	0.195	0.396	0.000	0.000	0.000
* LN(ASSETS)*	3,266	10.479	2.043	9.079	10.446	11.817
* ROA*	3,266	0.025	0.257	−0.015	0.047	0.131
* LEVERAGE*	3,266	0.189	0.264	0.008	0.076	0.267
* INTANGIBLES*	3,266	0.077	0.156	0.000	0.005	0.060
* LOSS*	3,266	0.317	0.465	0.000	0.000	1.000
* NON_US_ACQ*	7,534	0.348	0.477	0.000	0.000	1.000
Panel C: Acquirer-level descriptive statistics (U.S. Sample)						
Variables	N	Mean	SD	Q1	Median	Q4
* FOR_ACQ*	11,975	0.052	0.223	0.000	0.000	0.000
* POST*	11,975	0.218	0.413	0.000	0.000	0.000
* REPAT_TAX_COST*	11,975	0.442	0.497	0.000	0.000	1.000
* DOMESTIC*	11,362	0.410	0.492	0.000	0.000	1.000
* NON_INVGRADE_RATING*	11,791	0.866	0.340	1.000	1.000	1.000
* SALES_GROWTH*	11,975	0.138	0.458	−0.020	0.068	0.190
* WORKING_CAPITAL*	11,975	0.243	0.180	0.099	0.215	0.355
* LEVERAGE*	11,975	0.173	0.172	0.000	0.138	0.292
* MTB*	11,975	3.677	4.424	1.364	2.340	4.104
* SIZE*	11,975	6.421	2.212	4.881	6.486	7.969
* NOL*	11,975	0.728	0.445	0.000	1.000	1.000

This table presents the descriptive statistics for the global sample and the U.S. sample. The global sample includes all cross-border deals completed between 2011 and 2019. The U.S. sample includes all potential acquirers located in the United States. Panel A presents the composition of the global sample by target country. Panel B presents target-level descriptive statistics for the global sample. Panel C presents descriptive statistics for the potential acquirers included in the U.S. sample

#### U.S. sample of potential acquirers

We construct a U.S. sample by combining data on cross-border M&A from Zephyr with financial statement data from Compustat. We construct this sample in a way that allows us to test for the TCJA’s effect on the probability that a U.S. firm will acquire a foreign target and to examine whether this effect varies with the U.S. firm’s characteristics.

We first obtain a sample of firms incorporated in the United States with data available in Compustat for fiscal years 2010 to 2018.^20^ Following Hanlon et al. (2015), we exclude financial firms (SIC codes 6000–6999) and utilities (SIC codes 4900–4949). To facilitate the identification of firm-years affected by the TCJA, we drop observations with non-December fiscal year-ends (Beyer et al. 2022). Moreover, we drop firms with names ending in LP or TRUST, to exclude flow-through entities not subject to firm-level taxes (Dyreng et al. 2008). Consistent with prior research (Chay and Suh 2009; Hoberg et al. 2014), we delete observations with negative sales or negative total assets as well as those with book equity below $250,000 or total assets below $500,000. Finally, we drop those with insufficient data to compute our regression variables. Following these selection criteria results in a sample of 11,975 firm-year observations from Compustat.

In a final step, we merge the deal data with the Compustat sample. To this end, for each acquirer in our global sample, we determine whether its global ultimate owner is a U.S. firm. We then aggregate the deal-level data per global ultimate owner-year to obtain the number of foreign acquisitions by a U.S. global ultimate owner in year *t*. We also compute the annual value of these transactions. We link this data to the Compustat sample using the global ultimate owner’s International Securities Identification Number (ISIN), as reported in Orbis.^21^ In our final U.S. sample, 626 firm-years exhibit foreign acquisitions, representing 717 distinct deals. Panel C of Table 1 presents descriptive statistics for our U.S. sample. Overall we observe that approximately 5% of the firm-years in our U.S. sample report at least one acquisition of a foreign target.

## Main results

### Target-level analysis

#### Main specification

Panel A of Table 2 presents the main results of our target-level analysis. For all cross-border deals completed between 2011 and 2019, the likelihood that a target is acquired by a U.S. firm decreases after the TCJA, as indicated by the negative and significant coefficients on *POST* in columns (1) through (5).^22^ Across these columns, we employ various target-industry and target-country fixed effects. Including target-country-industry fixed effects in column (5) imposes the strictest design, capturing over-time variation in the probability of being acquired by a U.S. firm within each target-country-industry. In economic terms, the coefficients on *POST* in columns (4) and (5) indicate a decrease in the probability of being acquired by a U.S. firm of between 3.5 and 4.5 percentage points. Prior to the TCJA, the unconditional probability of being acquired by a U.S. firm, for foreign targets in our sample, is equal to 20.77%; our estimates imply a relative reduction by 16.8% to 21.7%. Descriptive analyses (untabulated) suggest that this reduction is due to *fewer* U.S. firms making a foreign acquisition rather than the same set of firms acquiring fewer targets.

**Table 2:** Target-level analysis

**Panel A: Main Results**						
	(1)	(2)	(3)	(4)	(5)	(6)
	Coef. (SE)	Coef. (SE)	Coef. (SE)	Coef. (SE)	Coef. (SE)	Coef. (SE)
Variables	*US_ACQ*	*US_ACQ*	*US_ACQ*	*US_ACQ*	*US_ACQ*	*NON_US_ACQ*
* POST*	−0.060***	−0.045***	−0.060***	−0.045***	−0.035**	0.010
	(0.016)	(0.016)	(0.016)	(0.016)	(0.017)	(0.012)
* LN(ASSETS)*	0.003	−0.003	0.004	−0.001	0.000	0.023***
	(0.003)	(0.003)	(0.003)	(0.003)	(0.004)	(0.003)
* ROA*	−0.047	−0.012	−0.042	−0.010	0.001	0.014
	(0.037)	(0.037)	(0.037)	(0.037)	(0.038)	(0.025)
* LEVERAGE*	−0.042	−0.009	−0.041	−0.009	−0.010	−0.016
	(0.027)	(0.026)	(0.027)	(0.027)	(0.029)	(0.023)
* INTANGIBLES*	0.171***	0.108**	0.157***	0.081*	0.050	0.168***
	(0.048)	(0.049)	(0.049)	(0.049)	(0.052)	(0.042)
* LOSS*	−0.013	−0.017	−0.007	−0.004	0.001	0.007
	(0.018)	(0.018)	(0.018)	(0.018)	(0.019)	(0.013)
* Intercept*	0.179***	0.235***	0.168***	0.216***	0.201***	0.104***
	(0.035)	(0.037)	(0.036)	(0.037)	(0.041)	(0.026)
Observations	3,266	3,266	3,266	3,266	3,208	7,534
Industry-FE	No	No	Yes	Yes	No	Yes
Country-FE	No	Yes	No	Yes	No	Yes
Country-Industry-FE	No	No	No	No	Yes	No
R^2^	0.009	0.082	0.021	0.097	0.149	0.229
**Panel B: Robustness Tests**						
	(1)		(2)		(3)	
	Coef. (SE)		Coef. (SE)		Coef. (SE)	
Variables	*US_ACQ*		*US_ACQ*		*US_ACQ*	
* POST*	−0.021***		−0.047***		−0.037*	
	(0.007)		(0.017)		(0.020)	
* LN(ASSETS)*	0.004***		−0.002		0.007	
	(0.001)		(0.004)		(0.005)	
* ROA*	−0.005		0.006		0.011	
	(0.016)		(0.040)		(0.046)	
* LEVERAGE*	−0.013		0.005		−0.018	
	(0.013)		(0.029)		(0.038)	
* INTANGIBLES*	0.086***		0.077		0.104	
	(0.027)		(0.053)		(0.068)	
* LOSS*	−0.005		−0.000		0.004	
	(0.008)		(0.020)		(0.026)	
* Intercept*	0.036**		0.220***		0.112**	
	(0.015)		(0.040)		(0.054)	
Observations	8,175		2,844		1,481	
Industry-FE	Yes		Yes		Yes	
Country-FE	Yes		Yes		Yes	
Country-Industry-FE	No		No		No	
R^2^	0.073		0.096		0.114	

This table presents regression results for the effect of the TCJA on the acquisition of foreign targets. Panel A presents the main results. Panel B presents results for robustness tests. In panel A (B), the samples in columns 1–5 (2–3) include cross-border acquisitions only; the sample in column 6 (1) includes cross-border acquisitions and domestic acquisitions, respectively. The sample in column 6 of panel A is limited to acquisitions by non-U.S. firms. All samples in panel A include acquisitions completed between 2011 and 2019. In panel B, the sample in column 1 (2) [3] includes acquisitions completed between 2011 and 2019 (excludes acquisitions completed in 2017) [is limited to acquisitions completed between 2016 and 2019]. In both panels, the dependent variable is an indicator variable equal to one if a target is acquired by a U.S. firm and zero otherwise (i.e., a target is acquired by a non-U.S. firm). In column 6 of panel A, the dependent variable is an indicator variable equal to one if a target is acquired by a foreign non-U.S. firm and zero otherwise (i.e., a target is acquired by a domestic firm located in the target country). The independent variables are lagged by one year. All regressions are estimated as linear probability models. The regression in column 2 (3) [5] of panel A includes target-industry (target-country) [target-country-industry] fixed effects. In panel A (B), the regressions in columns 4 and 6 (1–3) include target-country and target-industry fixed effects. We report heteroscedasticity-robust standard errors. *, **, and *** represent significance levels of 10%, 5%, and 1%, respectively (two-tailed)

In column (6), we modify our approach to explore whether the TCJA changed incentives for non-U.S. firms to make foreign acquisitions. Specifically, we modify the sample to remove deals involving U.S. acquirers and add domestic acquisitions, that is, when both acquirer and target are in the same country. Hence the coefficient on *POST* tells us whether the TCJA changed the probability of a deal occurring that involves a non-U.S. foreign acquirer, relative to a domestic acquirer. We fail to find evidence that the TCJA changed the incentives for non-U.S. firms to pursue cross-border M&A.^23^

Panel B of Table 2 offers several tabulated results from robustness tests. Specifically, column (1) expands our initial sample to include domestic acquisitions. Column (2) excludes all deals consummated in 2017, the year the TCJA was passed, to address concerns that U.S. firms’ foreign M&A changed in anticipation of the reform. Column (3) restricts the pre-reform period to 2016 and 2017, to see whether acquisition patterns between 2011 and 2015 drive our inferences. The takeaway from Table 2 is that the TCJA weakened the incentives of U.S. firms to acquire abroad.

Table 3 presents the results of cross-sectional tests in which we examine whether specific provisions of the TCJA changed U.S. firms’ incentives to acquire certain types of foreign targets. As discussed in Section 2.2, GILTI created a disincentive to earn excess profits in low-tax jurisdictions by imposing an immediate U.S. tax without regard to repatriation. As the precise minimum tax rate that will subject foreign income to GILTI varies across U.S. firms, we split our sample into relatively high- and low-taxed targets, bifurcating the sample at the annual median based on the target country’s statutory corporate income tax rate.^24^ In columns (1) and (2), we find evidence that the reduced likelihood of being acquired by a U.S. firm is concentrated in low-taxed foreign targets.^25^

**Table 3:** Target-level analysis (cross-sectional evidence)

	(1)	(2)	(3)	(4)	(5)	(6)
	Coef. (SE)	Coef. (SE)	Coef. (SE)	Coef. (SE)	Coef. (SE)	Coef. (SE)
Variables	*US_ACQ*	*US_ACQ*	*US_ACQ*	*US_ACQ*	*US_ACQ*	*US_ACQ*
Samples	Low tax rate	High tax rate	High profitability & Low tax rate	Remaining sample	Low sales growth	High sales growth
*POST*	−0.052**	−0.007	−0.080**	−0.024	−0.060***	−0.015
	(0.022)	(0.030)	(0.034)	(0.019)	(0.023)	(0.026)
*LN(ASSETS)*	−0.002	−0.001	−0.001	0.000	0.003	−0.004
	(0.004)	(0.006)	(0.007)	(0.004)	(0.006)	(0.006)
*ROA*	−0.006	−0.021	0.044	−0.032	0.061	−0.016
	(0.045)	(0.063)	(0.116)	(0.041)	(0.062)	(0.065)
*LEVERAGE*	−0.010	−0.006	0.036	−0.012	−0.047	−0.029
	(0.032)	(0.047)	(0.066)	(0.030)	(0.039)	(0.043)
*INTANGIBLES*	0.140**	−0.011	0.103	0.052	0.116	0.049
	(0.069)	(0.070)	(0.105)	(0.056)	(0.080)	(0.079)
*LOSS*	−0.019	0.007	0.000	−0.000	0.024	0.006
	(0.022)	(0.029)	(0.000)	(0.019)	(0.027)	(0.034)
*Intercept*	0.203***	0.236***	0.207**	0.191***	0.137**	0.258***
	(0.046)	(0.062)	(0.085)	(0.046)	(0.063)	(0.064)
p-Value (*POST*)	(1) < (2): 0.115		(3) < (4): 0.075		(5) < (6): 0.098	
Observations	1,789	1,476	896	2,368	1,248	1,242
Industry-FE	Yes	Yes	Yes	Yes	Yes	Yes
Country-FE	Yes	Yes	Yes	Yes	Yes	Yes
R^2^	0.130	0.068	0.128	0.100	0.112	0.136

This table presents results for cross-sectional tests for the effect of the TCJA on the likelihood that a foreign target is acquired by a U.S. firm. The samples in all columns include cross-border acquisitions completed between 2011 and 2019. The sample in column 1 (2) includes acquisitions in target countries with a statutory corporate income tax rate below (above) the annual sample median. The sample in column 3 (4) includes targets with profitability above the annual sample median and a statutory corporate income tax rate below the annual sample median (the remaining global sample). We measure profitability as the return on tangible fixed assets (based on EBIT) in the year prior to the deal. The sample in column 5 (6) includes targets with sales growth below (above) the annual sample median in the year prior to the deal. The dependent variable is an indicator variable equal to one if a target is acquired by a U.S. firm and zero otherwise (i.e., a target is acquired by a non-U.S. firm). The independent variables in all columns are lagged by one year. All regressions are estimated as linear probability models. All regressions include target-country and target-industry fixed effects. We report heteroscedasticity-robust standard errors. We estimate a fully-interacted model to assess whether the coefficients on *POST* differ between subsamples (Allison 1999). *, **, and *** represent significance levels of 10%, 5%, and 1%, respectively (two-tailed)

We tighten our tests surrounding the GILTI provision further by considering whether the low-taxed income expected to be generated by the target would be considered excess or intangible under GILTI. As discussed in Section 2.2, GILTI defines intangible income as that which exceeds a 10% return on tangible property. Accordingly, in columns (3) and (4), we bifurcate our sample of foreign targets at *both* the annual median tax rate (as in columns (1) and (2)) and the annual median profitability. In line with the GILTI provision, we define profitability using the return on tangible fixed assets.^26^ We find evidence that the reduced likelihood of foreign targets being acquired by a U.S. firm is concentrated in targets expected to generate specifically what GILTI calls *intangible* low-taxed income.^27^ Taken together, the results in columns (1) through (4) are consistent with the conclusion that GILTI discourages U.S. firms from acquiring profitable low-taxed foreign targets.

We test for an effect of the repeal of the repatriation tax in columns (5) and (6). As discussed in Section 2.2, the TCJA’s repeal of the U.S. repatriation tax reduced the tax disadvantage that U.S. firms had, as owners of foreign targets, relative to non-U.S. firms. However, eliminating the lockout effect also removed an internal capital market friction, making the repatriation of foreign earnings less costly and increasing the opportunity cost of investing abroad (Albertus et al. 2022). We therefore expect U.S. firms to become less likely to pursue low-growth investment projects abroad after the reform that might have attracted U.S. acquirers prior to the TCJA. When we split the sample at the annual median of target-level sales growth (Badertscher et al. 2013; Biddle et al. 2009), we find support for this conjecture. That is, the reduction in the likelihood of being acquired by a U.S. firm is stronger for low-growth targets with potentially limited investment opportunities (column (5)). We find consistent results when splitting the sample based on target-country GDP growth (untabulated).

#### Alternative specification

Our target-level analysis is a pre-post comparison of the probability that a foreign target is acquired by a U.S. firm. It thus does not allow us to compare the trends in foreign M&A of U.S. acquirers to those of non-U.S. acquirers. To strengthen our inferences in this regard, we apply an alternative empirical strategy based on the work of Feld et al. (2016). In a difference-in-differences conditional-logit framework, we test whether the likelihood that the *acquirer* of a foreign target is in the U.S. (treatment group), relative to the likelihood that the acquirer resides elsewhere (control group), changed in response to the TCJA.

For this setup, we duplicate the observations in our sample so that the acquirer could be in any acquirer country represented by the global sample. The dependent variable, *ACQ_COUNTRY*, is an indicator variable equal to one for the actual acquirer country and zero for all other countries in which the acquirer is *not* located. As an independent variable, we include the indicator variable *REFORM*, which is equal to one for the United States and zero for all other potential acquirer countries. We also include the variable *POST* and interact *REFORM* with *POST* to yield the difference-in-differences design.^28^ This design allows us to test whether the likelihood that the acquirer of target *i* is located in the United States (the treatment group) changed in response to the TCJA, relative to the likelihood that the acquirer is located in any other country (the control group). By including a fixed effect for each potential acquirer country, we exploit within-country variation in the taxation of potential acquirers located in the United States. In line with our target-level analysis, we expect a negative coefficient on REFORM*POST, indicating a lower likelihood that the acquirer of foreign target *i* is located in the United States post TCJA.

**Table 4:** Alternative specification

	(1)	(2)
	Coef. (SE)	Coef. (SE)
Variables	*ACQ_COUNTRY*	*ACQ_COUNTRY*
REFORM*POST	−0.327***	−0.352***
	(0.122)	(0.134)
* LN(GDP_CAPITA)*	−0.415	−0.109
	(0.405)	(0.420)
* GDP_GROWTH*	−0.011	−0.017
	(0.014)	(0.016)
* NUMBER_ACQUISITIONS*	0.192***	0.271***
	(0.023)	(0.044)
* LN(DISTANCE)*	−0.107***	−0.122***
	(0.035)	(0.045)
* NEIGHBORING*	0.708***	0.669***
	(0.082)	(0.103)
* COMM_LANGUAGE*	0.724***	0.566***
	(0.074)	(0.079)
* COLONY*	0.327***	0.297***
	(0.070)	(0.097)
* SAME_COUNTRY*	0.657***	0.222
	(0.185)	(0.225)
* MARKET_VALUE_EQUITY*		0.002
		(0.002)
* EXCHANGE_RATE*		0.001
		(0.001)
Observations	189,589	103,202
Country-FE	Yes	Yes
Pseudo R^2^	0.267	0.278

This table presents regression results for the effect of the TCJA on the likelihood that the acquirer of a foreign target is located in the United States. The samples in all columns include cross-border acquisitions completed between 2011 and 2019. The dependent variable is an indicator variable equal to one for the actual acquirer country and zero otherwise. All regressions are estimated as conditional logit models. All regressions include a fixed effect for each potential acquirer country in our global sample. We report heteroscedasticity-robust standard errors. *, **, and *** represent significance levels of 10%, 5%, and 1%, respectively (two-tailed)

Note that *REFORM* is collinear with the acquirer-country fixed effect and therefore subsumed in the regression. Moreover, the conditional-logit framework is based on a *within estimator* and leverages variation within each deal to estimate the likelihood that the acquirer of foreign target *i* is located in a given country. As a result, variables that do not vary across potential acquirer countries for a given target, such as *POST* or target-firm and target-country characteristics, are also subsumed in the estimation. As expected, the coefficient on REFORM*POST is negative and significant in column (1) of Table 4. We find consistent results when including additional controls in column (2) or excluding year 2017 observations (untabulated). In sum, these results corroborate the findings from our target-level analysis and provide additional evidence that U.S. firms are less dominant in the global M&A market after the TCJA.

**Fig. 2:**
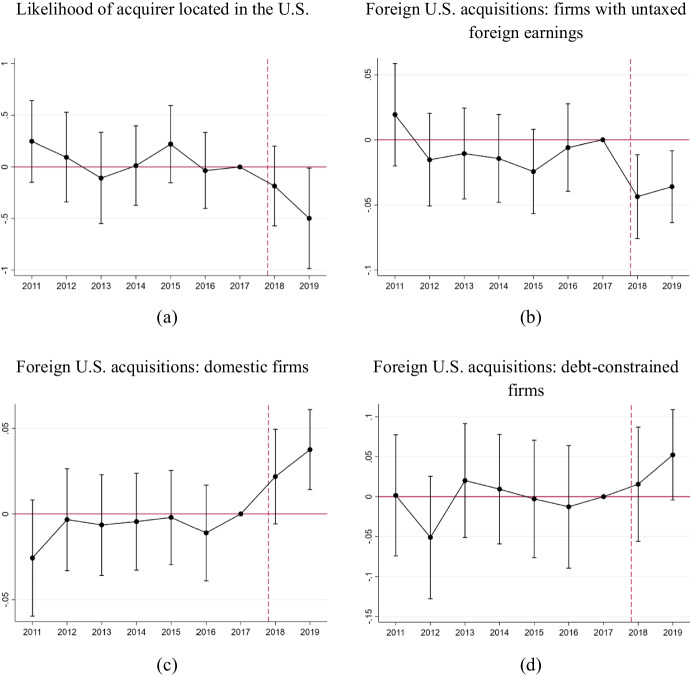
Yearly treatment effects. This figure shows yearly treatment effects. Part a presents results for the likelihood that the acquirer of a foreign target is located in the United States. Part b (c) [d] presents results for the likelihood that a U.S firm acquires a foreign target, where year indicators are interacted with *REPAT_TAX_COST* (*DOMESTIC*) [*NON_INVGRADE_RATING*]. Part a is based on a conditional logit model, while parts c–d are based on a linear probability model. The samples for all parts include cross-border acquisitions completed between 2011 and 2019. The coefficient estimates in all parts are constrained to zero for the year 2017. Hence yearly treatment effects have to be interpreted relative to this base year. The dotted red line marks the event of the tax reform. Whisker bars represent 95% confidence intervals

The approach in Table 4 also allows us to test for parallel pre-reform trends in the foreign M&A of U.S. and non-U.S. acquirers. To do so, we replace *POST* with a set of year indicators and estimate yearly treatment effects. We constrain the estimate to zero for the year 2017 (i.e., the year the TCJA passed) and estimate treatment effects, relative to this base year. We re-estimate the model in column (2) of Table 4 and depict our results in Fig. 2a. As is evident, yearly treatment effects in the pre-reform period are insignificant and vary unsystematically around zero (all p > 0.22). Further, these estimates are jointly insignificant (p = 0.53), and their sum is insignificantly different from zero (p = 0.63). For the post-reform period, we observe consistently negative treatment effects; these are strongest in 2019 (p = 0.05) and slightly less pronounced in 2018 (p = 0.35). This lag is reasonable, because cross-border deals take time, delaying a potential response to the TCJA. In sum, Fig. 2a indicates parallel trends in acquirer location prior to the TCJA. It suggests that differential pre-reform trends in the M&A of U.S. and non-U.S. acquirers do not drive our results.

To address concerns that other, non-TCJA related events in the United States or concurrent events in other major acquirer countries could drive our findings, we conduct two sets of untabulated placebo tests. First, we drop all post-reform observations and assume pseudo reforms in the United States for the years 2011 through 2016. When we re-estimate the regressions in columns (1) and (2) of Table 4 for each pseudo reform, the coefficients on REFORM*POST are all insignificant (all p > 0.26). Second, we drop all U.S. observations and assume a 2017 pseudo reform for each of the remaining top 10 acquirer countries in our sample.^29^ The coefficients on REFORM*POST are again insignificant for all pseudo reforms (all p > 0.30). In sum, these tests support the notion that the firms in our sample indeed responded to the TCJA and rule out that acquirers located in countries other than the United States or pre-reform events in the United States drive our findings.^30^

### Acquirer-level analysis

Next we focus on potential U.S. acquirers and examine their changing propensity to purchase a foreign target due to the TCJA. As noted in Section 3.1.2, we identify heterogeneous responses to the reform based on the pre-reform characteristics of the potential acquirers in our U.S. sample. We specifically examine three firm characteristics measured using indicator variables: i) whether the U.S. firm had untaxed foreign earnings prior to the TCJA (*REPAT_TAX_COST*), ii) whether the U.S. firm had a significant foreign presence prior to the TCJA (*DOMESTIC*), and iii) whether the U.S. firm had no or a non-investment grade credit rating prior to the TCJA (*NON_INVGRADE_RATING*). We report the results in Table 5.^31^

**Table 5:** Acquirer-level analysis (cross-sectional evidence)

	(1)	(2)	(3)
	Coef. (SE)	Coef. (SE)	Coef. (SE)
Variables	*FOR_ACQ*	*FOR_ACQ*	*FOR_ACQ*
REPAT_TAX_COST*POST	−0.032***		
	(0.009)		
DOMESTIC*POST		0.036***	
		(0.008)	
NON_INVGRADE_RATING*POST			0.038*
			(0.021)
* SALES_GROWTH*	0.004	0.006*	0.004
	(0.003)	(0.003)	(0.003)
* WORKING_CAPITAL*	0.021	0.030	0.021
	(0.026)	(0.026)	(0.026)
* LEVERAGE*	−0.039*	−0.037	−0.040*
	(0.021)	(0.023)	(0.021)
* MTB*	0.002***	0.001**	0.002***
	(0.001)	(0.001)	(0.001)
* SIZE*	−0.012**	−0.016***	−0.013**
	(0.006)	(0.006)	(0.006)
* NOL*	−0.011	−0.011	−0.010
	(0.008)	(0.008)	(0.008)
* Intercept*	0.136***	0.155***	0.128***
	(0.038)	(0.040)	(0.039)
Observations	11,975	11,362	11,791
Firm-FE	Yes	Yes	Yes
Year-FE	Yes	Yes	Yes
R^2^	0.250	0.251	0.249

This table presents results for the effect of the TCJA on the likelihood that a U.S. firm acquires a foreign target, conditional on the characteristics of the potential U.S. acquirer. The samples in all columns include foreign acquisitions of U.S. firms completed between 2011 and 2019. The dependent variable is an indicator variable equal to one if a U.S. firm acquires a foreign target in year *t* and zero otherwise (i.e., a U.S. firm does not acquire a foreign target in year *t*). The independent variables in all columns are lagged by one year. All regressions are estimated as linear probability models. All regressions include firm and year fixed effects. We report heteroscedasticity-robust standard errors, clustered by firm. *, **, and *** represent significance levels of 10%, 5%, and 1%, respectively (two-tailed)

As expected, the point estimate on REPAT_TAX_COST*POST in column (1) suggests that a firm with untaxed foreign earnings, on average, exhibits a 3.2 percentage point *lower* probability of acquiring a foreign target after the passage of the TCJA than does a firm without untaxed earnings.^32^ Prior to the TCJA, untaxed foreign earnings represented, to a large extent, trapped cash. After the TCJA, cash can be used for investment at home or abroad with an equal tax cost of doing so. As firms can now repatriate this cash at no additional cost, the TCJA increases the opportunity cost of investing abroad (Edwards et al. 2016; Albertus et al. 2022) and thus reduces the likelihood that foreign cash will be used to acquire foreign targets. Hence the repeal of the repatriation tax helped level the playing field with respect to investment opportunities for foreign cash. However, concurrent research investigating other potential firm responses to the TCJA finds little evidence for changes in domestic investment (Beyer et al. 2022). Rather, U.S. firms whose foreign cash is no longer trapped after the reform tend to increase dividend payouts and share repurchases and thus distribute the freed-up cash to their shareholders (Beyer et al. 2022; Olson 2021; Bennett and Wang 2021).^33^ We test for an effect of the TCJA on domestic acquisitions in Section 5.3.

Again we also estimate yearly treatment effects for the model in column (1) of Table 5 to assess whether treatment and control firms exhibit parallel pre-reform trends in the likelihood of acquiring a foreign target. In Fig. 2b, the yearly treatment effects are insignificant pre-reform (all p > 0.14). The estimates are also jointly insignificant (p = 0.35), and their sum is not significantly different from zero (p = 0.54), suggesting parallel pre-reform trends in the outbound M&A. Untabulated tests reveal that the result in column (1) is also robust to excluding deals completed in 2017, examining the quartile rank of untaxed foreign earnings rather than the existence, measuring repatriation tax costs using required firm-level disclosures of the TCJA transition tax, and using a shorter pre-reform period (2016 and 2017).^34^ Moreover, we find no change in the annual number of deals per firm.^35^ Thus our results are consistent with fewer U.S. firms with untaxed foreign earnings making a foreign acquisition post TCJA.

The point estimate on DOMESTIC*POST in column (2) implies that a U.S. firm without a significant foreign presence prior to the passage of the TCJA exhibits a 3.6 percentage point *higher* probability of acquiring a foreign target after the reform than does a multinational firm. Figure 2c indicates that treatment and control firms again exhibit similar pre-reform trends in their probability of acquiring a foreign target.^36^ Our result is consistent with the TCJA inducing firms without a significant history of foreign operations to expand abroad. The repeal of the U.S. repatriation tax on future foreign income reduced the marginal cost of funding foreign acquisitions with domestic funds (Liu 2020), making foreign acquisitions more attractive. At the same time, the reduction in the U.S. statutory corporate income tax rate generated cash-tax savings, increasing the domestic funds available for foreign investment. In column (3), the coefficient on NON_INVGRADE_RATING*POST indicates that U.S. firms with limited access to public debt markets exhibit a 3.8 percentage point *higher* probability of acquiring a foreign target than does a firm whose access is less constrained.^37^ The lower U.S. statutory corporate income tax rate after the TCJA generates cash-tax savings that increase internal funds available for foreign investment; this increase is particularly beneficial for debt-constrained firms whose investment decisions are sensitive to internal cash flow.^38^ The results in columns (2) and (3) are again robust to excluding acquisitions completed during 2017 and using a shorter pre-reform period (2016 and 2017). In both tests, we find no change in the annual number of completed deals per firm, consistent with the TCJA incentivizing more U.S. firms to expand abroad.

To further corroborate our finding that the TCJA overall damped the foreign M&A of potential U.S acquirers, in untabulated tests, we expand our U.S. sample to include both U.S. and Canadian firms. In a sample of potential acquirers from both countries, we may identify the overall shift in the likelihood that U.S. firms acquire a foreign target, relative to Canadian firms. We choose Canadian firms as a control group because these firms are economically comparable to U.S. firms while not being directly affected by the TCJA.^39^ In specifications with and without firm and year fixed effects, we find evidence consistent with a decline in the probability that a U.S. firm acquires a foreign target, relative to Canadian firms.^40^ The foreign M&A of Canadian firms, however, did not change in response to the TCJA. Overall these findings are consistent with the results discussed earlier from panel A of Table 2 (column (6)), indicating that the TCJA did not change the overall incentives for non-U.S. firms to pursue cross-border M&A but did decrease the foreign M&A of U.S. firms.

## Additional analyses

### Deal announcement returns

As discussed in Section 2.2, the U.S. repatriation tax was abolished to address the lock-out effect, encouraging firms to repatriate their foreign earnings without tax friction. Prior to the TCJA, Hanlon et al. (2015) found that U.S. firms with a greater accumulation of foreign cash, due to repatriation tax avoidance, were more likely to acquire abroad. However, due to potential agency conflicts over how to employ foreign cash (Amberger et al. 2021), investors discounted the valuations of these deals.

In Table 6, we examine deal announcement returns for periods both before and after the tax reform. In columns (1) and (2), we find that deal announcement returns for firms with higher repatriation tax costs (and thus a greater accumulation of foreign cash) are relatively higher after the TCJA, as indicated by the positive coefficient on REPAT_TAX*POST.^41^ The coefficient on *REPAT_TAX* in the period prior to the TCJA is negative (consistent with Hanlon et al. 2015) but insignificant. Our results are stronger in columns (3) and (4), when we eliminate deals announced during the U.S. election year (2016) and the year of the tax reform (2017). Collectively, these results suggest the TCJA eliminated a tax friction, allowing firms to effectuate more value-enhancing deals (in expectation) with less potential agency costs.

**Table 6:** TCJA and deal announcement returns

	(1)	(2)	(3)	(4)
	Coef. (SE)	Coef. (SE)	Coef. (SE)	Coef. (SE)
Variables	*CAR*	*CAR*	*CAR*	*CAR*
* REPAT_TAX*	−0.029	0.142	−0.138	−0.027
	(0.199)	(0.215)	(0.212)	(0.193)
REPAT_TAX*POST	0.733*	0.570	0.899**	0.773**
	(0.370)	(0.410)	(0.275)	(0.308)
* LEVERAGE*	0.040	0.038	0.038	0.036
	(0.023)	(0.022)	(0.022)	(0.023)
* MTB*	−0.001**	−0.001*	−0.002***	−0.001**
	(0.001)	(0.001)	(0.000)	(0.000)
* SIZE*	−0.003	−0.002*	−0.003	−0.003
	(0.001)	(0.001)	(0.002)	(0.001)
* LN(DEAL_VALUE)*	0.004*	0.003	0.004	0.004
	(0.002)	(0.002)	(0.002)	(0.002)
* DIVERSIFYING*	0.005	0.006	0.004	0.006
	(0.005)	(0.005)	(0.004)	(0.003)
* PUBLIC_TARGET*	−0.017*	−0.018**	−0.017	−0.016
	(0.008)	(0.007)	(0.010)	(0.009)
* Intercept*	−0.013	−0.015	−0.008	−0.011
	(0.016)	(0.018)	(0.020)	(0.022)
Observations	733	733	589	589
Industry-FE	No	Yes	No	Yes
Country-FE	No	Yes	No	Yes
Year-FE	Yes	Yes	Yes	Yes
R^2^	0.049	0.111	0.054	0.118

This table presents results for announcement-return tests, conditional on the repatriation tax costs of a U.S. acquirer. The samples in columns 1–2 include foreign acquisitions of U.S. firms announced between 2011 and 2019. The samples in column 3–4 exclude acquisitions announced in the years 2016 or 2017. The dependent variable is the cumulative abnormal return of a U.S. acquirer, computed for a five-day window around the announcement of the foreign acquisition (*t−2* to *t* + *2*). Acquirer-level independent variables in all columns are lagged by one year. All regressions are estimated as linear regression models. The regressions in columns 1 and 3 (2 and 4) include year (target-industry, target-country, and year) fixed effects. We report heteroscedasticity-robust standard errors, clustered by firm and year. *, **, and *** represent significance levels of 10%, 5%, and 1%, respectively (two-tailed)

### Cash versus noncash acquisitions

We argued in Section 2.2 that the lower U.S. statutory corporate income tax rate after the TCJA generates cash-tax savings that U.S. firms can spend on foreign acquisitions. As a result, we would expect a larger share of U.S. acquisitions to be financed with cash after the reform. To test this prediction, we collect information on the deal payment method from Zephyr and perform several univariate and multivariate tests (untabulated). We obtain payment method for 1,584 of the 3,266 deals in the global sample. Nine hundred and seventeen of these deals are fully financed with cash (cash deals); another 134 are fully financed with stock (noncash deals).

We observe in these data that the share of foreign cash, relative to noncash, acquisitions increased after the TCJA (p = 0.04). This increase is driven by deals with a U.S. acquirer (p = 0.03); the share of cash acquisitions did not significantly change for non-U.S. deals (p = 0.23). We corroborate this result in a multivariate analysis where we replace the dependent variable in Eq. (1) with an indicator variable equal to one for a cash acquisition and zero for a noncash acquisition. When estimating the resulting regression separately for U.S. and non-U.S. acquisitions, we find an increase in the likelihood of a cash acquisition for U.S. deals post TCJA (p < 0.01) while the likelihood did not change for non-U.S. deals (p = 0.72). Collectively, these results suggest that cash acquisitions abroad became more common among U.S. acquirers after the reform, consistent with the argument that the TCJA provides potential U.S. acquirers with tax-cash savings that some of them spend on foreign acquisitions.

### Domestic U.S. acquisitions

Our main analyses are primarily aimed at better understanding how the TCJA changed incentives for U.S. firms to acquire *abroad*. However, the TCJA also provides an opportunity to extend the analysis of Bird et al. (2017), which examines *domestic* acquisitions. Policymakers noted that another competitive concern surrounding the pre-TCJA U.S. international tax system was that U.S. firms were disproportionately targets for acquisition by foreign firms. Bird et al. (2017) found evidence consistent with foreign firms resident in countries with a territorial tax system being tax-favored acquirers of U.S. targets, particularly U.S. targets with large untaxed foreign earnings. To see whether the TCJA removed this disadvantage for U.S. acquirers of U.S. targets, we re-estimate our target-level analysis on a sample of U.S. deals and examine the effect of the TCJA on the probability that U.S. target *i* is acquired by a U.S. firm.^42^ Since this analysis focuses on U.S. deals only, we do not include target-country fixed effects.

We present the results in Table 7. In column (1), we find a positive and significant coefficient on *POST*. This result indicates that the TCJA had a positive effect on U.S. acquisitions by U.S. firms, due to reducing the tax advantage of foreign bidders. Results are qualitatively similar when we eliminate deals completed in 2017 and when we limit the pre-reform period to the years 2016 and 2017 (untabulated). To further tighten this analysis, we consider specific attributes of U.S. targets that would make them relatively more attractive to U.S. acquirers after the tax reform. In columns (2) and (3), we partition the sample of U.S. targets into those with and without untaxed foreign earnings as of the date of the acquisition. We find that the positive effect is stronger for targets with untaxed foreign earnings, consistent with the elimination of U.S. tax on their *future* foreign earnings making them more attractive for U.S. acquirers.^43^ In columns (4) and (5), we partition the sample of U.S. targets into those more and less likely to benefit from the FDII regime. As discussed in Section 2.2 and Appendix 1, the FDII provisions of the TCJA offer a reduced tax rate on excess U.S. corporate profits earned from serving foreign markets. Thus we identify targets most likely to benefit from FDII as those reporting high profits (above the annual median return on tangible assets) and non-U.S. sales.^44^ We find that the increase in the probability of being acquired by a U.S. firm is stronger for these targets, consistent with the intent of the FDII provisions.

**Table 7:** TCJA and U.S. acquisitions of U.S. targets

	(1)	(2)	(3)	(4)	(5)
	Coef. (SE)	Coef. (SE)	Coef. (SE)	Coef. (SE)	Coef. (SE)
Variables	*US_ACQ*	*US_ACQ*	*US_ACQ*	*US_ACQ*	*US_ACQ*
Samples	Full sample	Untaxed foreign earnings	No untaxed foreign earnings	High profitability & non-U.S. sales	Remaining sample
* POST*	0.052*	0.150**	0.014	0.227*	0.012
	(0.032)	(0.075)	(0.035)	(0.120)	(0.048)
* LN(MARKET_CAP)*	−0.004	0.013	−0.008	0.087**	0.004
	(0.008)	(0.020)	(0.009)	(0.035)	(0.019)
* ROA*	0.038	−0.339	0.063	−1.161	0.214
	(0.117)	(0.260)	(0.131)	(1.077)	(0.344)
* LEVERAGE*	0.019	−0.003	−0.002	−0.041	0.218*
	(0.051)	(0.131)	(0.057)	(0.328)	(0.120)
* INTANGIBLES*	−0.117*	−0.146	−0.028	−0.030	−0.333
	(0.071)	(0.157)	(0.079)	(0.325)	(0.216)
* LOSS*	−0.002	−0.000	−0.014	0.000	0.070
	(0.041)	(0.110)	(0.044)	(0.000)	(0.093)
* Intercept*	0.860***	0.690***	0.920***	0.106	0.720***
	(0.063)	(0.152)	(0.069)	(0.380)	(0.161)
p-Value (*POST*)	-	(2) > (3): 0.048		(4) > (5): 0.033	
Observations	850	230	612	59	162
Industry-FE	Yes	Yes	Yes	Yes	Yes
R^2^	0.050	0.049	0.081	0.304	0.161

This table presents regression results for the effect of the TCJA on the likelihood that a U.S. target is acquired by a U.S. firm. The samples in all columns include acquisitions completed between 2011 and 2019. The sample in column 2 (3) includes targets with repatriation tax costs (no repatriation tax costs) prior to the acquisition. The sample in column 4 (5) includes targets with non-U.S. sales and profitability above the annual sample median (the remaining target sample). We measure profitability as the return on property, plant, and equipment (based on EBIT) in the year prior to the deal. The dependent variable is an indicator variable equal to one if a target is acquired by a U.S. firm and zero otherwise (i.e., a target is acquired by a non-U.S. firm). The independent variables in all columns are lagged by one year. All regressions are estimated as linear probability models. All regressions include target-industry fixed effects. We report heteroscedasticity-robust standard errors. We estimate a fully interacted model to assess whether the coefficients on *POST* differ between subsamples (Allison 1999). *, **, and *** represent significance levels of 10%, 5%, and 1%, respectively (two-tailed)

Finally, we test whether U.S. firms that reduced their cross-border M&A in response to the TCJA (i.e., firms with untaxed foreign earnings) changed their *domestic* acquisitions. As discussed in Section 4.2, concurrent research finds little evidence for domestic investment responses as U.S. firms whose foreign cash is no longer trapped after the reform tend to distribute the freed-up cash to their shareholders (Beyer et al. 2022; Olson 2021; Bennett and Wang 2021). To see whether the TCJA changed the domestic M&A of firms with untaxed foreign earnings, we re-estimate the acquirer-level analysis in column (1) of Table 5. Specifically, we replace the dependent variable with an indicator equal to one if firm *i* acquires at least one domestic target in year *t* and zero otherwise. In line with the findings in concurrent work, we find no change in domestic M&A (untabulated). Collectively, our results suggest that the decrease in outbound M&A did not facilitate acquisitions in the United States.

## Conclusion

Prior to the TCJA, the U.S. corporate tax system was perceived as distorting U.S. firms’ foreign investment decisions. Cross-border M&A patterns that regularly resulted in foreign ownership of U.S. assets were an oft-cited indicator that the U.S. international tax system was flawed (Lyon 2020). Not only were U.S. firms targeted for acquisition by foreign firms (Bird et al. 2017), they were also disadvantaged when bidding for foreign targets (Feld et al. 2016). This was primarily due to the high U.S. statutory corporate income tax rate of 35% and the U.S. tax levied on foreign-source income upon repatriation.

Signed into law on December 22, 2017, the TCJA introduced features of a territorial tax system (i.e., elimination of the U.S. repatriation tax), alongside features of a worldwide tax system (i.e., the GILTI regime). These changes were complemented by a substantial reduction in the U.S. statutory corporate income tax rate. We examine how the TCJA altered U.S. firms’ decisions to acquire foreign targets to determine whether and the to what extent the reform addressed the policy objectives to remove investment distortions for U.S. firms that prominently featured in political debates. Understanding the effects on firms’ incentives under this new hybrid system is imperative in light of the radical changes that were made to the U.S. tax system for the first time in three decades. We conclude that firms are responding as intended to the policy objectives of the TCJA, by increasing foreign acquisitions in some cases while decreasing them in other cases as well as changing the characteristics of foreign targets acquired.

Specifically, we document an overall decreased probability that a U.S. firm makes a foreign acquisition after the passage of the TCJA as well as both target and acquirer characteristics associated with differential responses to the TCJA’s key provisions. The TCJA’s most significant international provision was the repeal of the repatriation tax. Relatedly, we find a lower post-reform likelihood that U.S. firms with untaxed foreign earnings acquire a foreign target, a higher likelihood that U.S. firms with no international presence acquire a foreign target, and a decreased probability that U.S. firms acquire a low-growth foreign target. When examining deal announcement returns, we find that returns are relatively higher after the TCJA for deals announced by acquirers with large untaxed foreign earnings. Thus we conclude that, while the repeal of the repatriation tax both increased and decreased incentives for foreign M&A, firms with a lot of locked-out cash prior to the TCJA tend to pursue more value-enhancing acquisitions after the reform.

We also examine other key provisions of the TCJA. Most notably, we find that the GILTI regime reduces incentives for U.S. firms to acquire profitable low-taxed targets. Thus any further action by the Biden administration to strengthen GILTI would further disadvantage U.S. firms bidding for these targets. Future research should consider the anticipated adoption of OECD’s Pillar Two, a global minimum tax like the U.S. GILTI, on both the absolute and relative (to U.S. firms) incentives for outbound M&A. The Pillar Two rules are intended to be implemented as part of a common approach to taxing foreign income, as agreed by the OECD members, and to be brought into domestic legislation by 2023. However, each jurisdiction will need to determine when the rules would be enacted and effective. The Pillar Two global minimum tax would co-exist with the U.S. GILTI regime, as the United States is currently expected to keep the GILTI regime in place (Neubig 2020). This means that U.S. multinationals would be subject to GILTI while non-U.S. multinationals would be subject to the global minimum tax. Important differences between the U.S. GILTI and the Pillar Two global minimum tax adopted by other countries, such as the minimum tax rate and whether the tax is calculated on a global or jurisdictional basis, could make U.S. firms more or less competitive than non-U.S. firms when bidding for foreign targets.

## Data Availability

We obtain data from the public sources identified in the paper.

## Appendix 1

### Detailed discussion of pertinent TCJA provisions

The TCJA of 2017 is one of the most significant tax reforms the U.S. has experienced in decades, changing the incentives for many corporate decisions. One of the challenges for empiricists interested in studying its impact is that the law contains multiple important policy changes that cannot be viewed in isolation. The purpose of this appendix is to describe in detail each change that we expect to impact the incentives for outbound M&A by U.S. firms. In Fig. 1 and in Section 2.2 of the manuscript, we summarize these provisions and their expected effects on the acquisitions of U.S. firms.

#### Provision #1: change in the U.S. federal statutory tax rate for corporate income

One of the key domestic provisions in the TCJA was the reduction in the U.S. federal statutory corporate income tax rate from 35 to 21%. This change impacts all existing and potential U.S. operations, because it increases firms’ expected after-tax cash flows (Dyreng et al. 2020). Several other provisions (described below) can increase or decrease the U.S. effective tax rate for any given level of U.S. income, depending on certain characteristics of U.S. firms’ domestic and foreign operations. Also worth noting is that the corporate alternative minimum tax was repealed and there is no sunset provision, making the statutory corporation income tax rate reduction permanent.

#### Provision #2: one-time transition tax and future tax-free repatriation of foreign earnings

One of the key international provisions was the abolishment of the U.S. repatriation tax on future income earned abroad. Prior to the TCJA, a U.S. parent company faced a 35% U.S. corporate income tax (minus applicable foreign tax credits) on dividend distributions from its foreign earnings. As a result, U.S. multinationals generally preferred to retain their foreign earnings in no- or low-tax countries. Under the new system, a foreign subsidiary’s distribution of *future* earnings will no longer give rise to U.S. tax at the U.S. parent company. As part of the transition to this new system, the U.S. imposed a one-time tax on U.S. multinationals’ accumulated untaxed foreign earnings. The included income was subject to U.S. tax at the rate of 15.5% or 8%, with the latter, lower rate being applicable to noncash assets. The TCJA allowed U.S. multinationals to elect to pay this one-time transition tax in eight back-loaded annual installments and without incurring any interest charge. Even if this option were chosen, an immediate distribution of the accumulated foreign earnings would be tax-free and would not accelerate the tax liability.^45^ Overall the repeal of the repatriation tax was enacted to address the so-called lock-out effect whereby U.S. firms accumulated earnings and cash outside the United States to avoid the repatriation tax, encouraging repatriation and making the tax code more neutral to domestic versus foreign investment.

#### Provision #3: global intangible low-taxed income

The TCJA was heralded as incorporating territoriality into the U.S. tax system through provision #2 described above. Similar to the practices followed by other developed countries, income earned by foreign subsidiaries of U.S. firms would not be subject to U.S. taxation, either when earned or when distributed to the U.S. parent. In reality, the TCJA offers only “partial” or “quasi” territoriality, due to the introduction of the GILTI regime, which subjects some *active* foreign earnings to an immediate U.S. tax. The GILTI regime was introduced as an anti-abuse provision meant to prevent U.S. companies from more aggressively shifting income out of the United States to low-tax countries once the repatriation tax on future foreign earnings was removed.

In broad terms, GILTI operates in two ways. First, a foreign subsidiary’s earnings in excess of 10% of its depreciable foreign tangible property is considered to be intangible income and potentially subject to U.S. tax. Second, GILTI determines whether the income was low-taxed by reference to the effective tax rate paid in the foreign subsidiary’s host country. Assuming no underlying foreign income taxes were paid on such income, the effective U.S. tax rate on it is 10.5% for the taxable years beginning after December 31, 2017, and before January 1, 2026. Because of the interplay of revised foreign tax credit rules, the minimum foreign tax rate at which no U.S. tax would be due on such income is 13.125%. The minimum foreign tax rate increases to 16.406% for taxable years beginning after December 31, 2025. However, due to the complexity of U.S. expense allocation rules, these minimum rates can vary across firms. There is no sunset provision.

Thus GILTI becomes more onerous over time. As the foreign effective tax rate that triggers the GILTI tax rises, investment in low-tax countries should become less attractive because GILTI increases the tax cost of earning profits in low-tax jurisdictions. Further, since GILTI is tied to the excess return on a subsidiary’s tangible property, the provisions should discourage investment in intellectual property abroad and the acquisition of profitable targets. Also, the calculation of GILTI for any given U.S. multinational is aggregated over all of its foreign subsidiaries, which makes attempts by these multinationals to manipulate the GILTI rules challenging.

#### Provision #4: foreign-derived intangible income

Intended to attract cross-border investment back to the United States and incentivize U.S. businesses to operate domestically, the FDII provisions impose a tax rate lower than 21% on certain U.S. income derived from serving foreign customers. In broad terms, FDII also operates in two ways. First, a U.S. corporation’s earnings in excess of 10% of its depreciable U.S. tangible property is considered intangible income and is potentially eligible for the reduced U.S. tax rate. Second, the share of U.S. income related to the export of goods and services is determined as the share of the U.S. tax base eligible for the reduced rate.^46^ Thus FDII is intended to be a tax incentive to generate sizeable U.S. profits from serving foreign markets. As these profits are deemed to be related to the use of intellectual property, FDII is an attempt to reverse the intangible asset migration by U.S. firms over the past two decades.

The effective U.S. tax rate on such income is 13.125% (through a 37.5% deduction) for taxable years beginning after December 31, 2017, and before January 1, 2026. The effective U.S. rate increases to 16.406% (through a decrease in the deduction to 21.875%) for taxable years beginning after December 31, 2025. Thus FDII becomes less beneficial over time. There is no sunset provision. The EU has voiced concerns that FDII may violate international trade law. The U.S., however, argues that FDII is intended to work in tandem with GILTI to neutralize tax as a driver of where to locate intellectual property. Consequently, FDII may lower incentives to invest abroad and instead increase incentives to serve foreign markets through export sales.

#### Provision #5: base erosion and anti-abuse tax

To manage the erosion of the U.S. tax base, through payments by U.S. multinationals to their foreign affiliates giving rise to U.S. deductions, the base erosion and anti-abuse tax (BEAT) was added to the TCJA. BEAT applies to base erosion payments made or accrued in taxable years beginning after December 31, 2017, by U.S. corporations with average annual gross receipts of at least $500 million over the prior three-year period (aggregating related U.S. corporations and certain foreign subsidiaries) and a base erosion percentage generally of 3% or more.

BEAT is an add-on minimum tax and is due in any year in which it exceeds the regular tax liability of a U.S. corporation. The BEAT base is equal to the sum of the corporation’s regular tax base and, in general, the operating expenses paid by a U.S. corporation to its foreign affiliates that give rise to U.S. tax deductions. The BEAT rate is 5% for a taxable year beginning in 2018, 10% for taxable years beginning after December 31, 2018, and before January 1, 2026, and 12.5% for taxable years beginning after 2025. There is no reduction in the regular U.S. corporate tax liability in a future taxable year, making BEAT a permanent increase in the corporation’s effective tax rate.

## Appendix 2

**Table:** Variable Definitions

Variable	Description	Source
Target-Level Analysis (Global Sample)		
* US_ACQ*	Indicator variable equal to one if foreign target *i* is acquired by a firm that has a global ultimate owner located in the United States and zero otherwise	Zephyr Orbis
* POST*	Indicator variable equal to one if the deal involving foreign target *i* is completed after 2017 and zero otherwise	Zephyr
* LN(ASSETS)*	The natural logarithm of total assets of target *i* in the year prior to the deal	Orbis
* ROA*	Earnings before interest and taxes (EBIT) of target *i* in the year prior to the deal, scaled by total assets in the year prior to the deal	Orbis
* LEVERAGE*	Noncurrent liabilities of target *i* in the year prior to the deal, scaled by total assets in the year prior to the deal	Orbis
* INTANGIBLES*	Intangible fixed assets of target *i* in the year prior to the deal, scaled by total assets in the year prior to the deal	Orbis
* LOSS*	Indicator variable equal to one if the EBIT of target *i* in the year prior to the deal is negative and zero otherwise	Orbis
Additional Variables for the Global Sample		
* NON_US_ACQ*	Indicator variable equal to one if target *i* is acquired by a firm that has a foreign global ultimate owner located outside the United States and zero otherwise (i.e., target *i* is acquired by a firm that has a global ultimate owner located in the target country)	Zephyr Orbis
Alternative Empirical Strategy (Global Sample)		
* ACQ_COUNTRY*	Indicator variable equal to one for the country in which the ultimate owner of the firm that acquires foreign target *i* is located and zero for all other potential acquirer countries in the global sample	Zephyr Orbis
* REFORM*	Indicator variable equal to one for the United States as a potential acquirer country and zero otherwise	Zephyr Orbis
* LN(GDP_CAPITA)*	Natural logarithm of the GDP per capita in the potential acquirer country in the year prior to the deal	Worldbank
* GDP_GROWTH*	Annual GDP growth in the potential acquirer country in the year prior to the deal	Worldbank
* NUMBER_ACQUISITIONS*	Number of deals in the one-digit NACE industry of target *i* in the year prior to the deal with acquirers located in the potential acquirer country	Zephyr
* LN(DISTANCE)*	Natural logarithm of the simple distance between the country of target *i* and the potential acquirer country	CEPII
* NEIGHBORING*	Indicator variable equal to one if the country of target *i* and the potential acquirer country share a common border and zero otherwise	CEPII
* COMM_LANGUAGE*	Indicator variable equal to one if the country of target *i* and the potential acquirer country share a common language and zero otherwise	CEPII
* COLONY*	Indicator variable equal to one if the country of target *i* and the potential acquirer country were ever in a colonial relationship and zero otherwise	CEPII
* SAME_COUNTRY*	Indicator variable equal to one if the country of target *i* and the potential acquirer country were ever part of the same country and zero otherwise	CEPII
* MARKET_VALUE_EQUITY*	Market capitalization of listed domestic companies to GDP in the potential acquirer country in the year prior to the deal	Worldbank
* EXCHANGE_RATE*	National currency in the potential acquirer country in the year prior to the deal, expressed in U.S. dollar per national currency unit	OECD
Acquirer-Level Analysis (U.S. Sample)		
* FOR_ACQ*	Indicator variable equal to one if U.S. firm *i* acquires at least one foreign target in year *t* and zero otherwise	Zephyr Compustat
* SALES_GROWTH*	Sales growth of firm *i* in year *t−1*, as sales (SALE) in year *t−1* less sales (SALE) in year *t−2*, scaled by sales (SALE) in year *t−2*	Compustat
* WORKING_CAPITAL*	Working capital of firm *i* in year *t−1*, as total current assets (ACT), less debt in current liabilities (DLC), less cash and short-term investments (CHE), and scaled by total assets (AT)	Compustat
* LEVERAGE*	Leverage of firm *i* in year *t−1*, as long-term debt (DLTT), scaled by total assets (AT)	Compustat
* MTB*	Market-to-book ratio of firm *i* in year *t−1*, as market value of equity (PRCC*CSHO), scaled by stockholder’s equity (SEQ)	Compustat
* SIZE*	Size of firm *i* in year *t−1*, as the natural logarithm of total assets (AT)	Compustat
* NOL*	Indicator variable equal to one if firm *i* reports a tax loss carry forward (TLCF) in year *t−1* and zero otherwise. Missing values for TLCF are set to zero	Compustat
Partitioning Variables (U.S. Sample)		
* REPAT_TAX_COST*	Indicator variable equal to one if firm *i* has positive repatriation tax costs in the year 2016 and zero otherwise. We calculate *REPAT_TAX_COST* by taking the three-year average of *REPAT_TAX* for the years 2014–2016. In line with Foley et al. (2007), we compute *REPAT_TAX* in year *t* as pre-tax foreign income (PIFO) multiplied by 0.35 less foreign income taxes (TXFO). The difference is scaled by total assets (AT). Missing values for *REPAT_TAX* are set to zero	Compustat
* DOMESTIC*	Indicator variable equal to one if firm *i* is a domestic firm in the year 2016, and the value of zero if firm *i* is a multinational in the year 2016. We classify a firm as domestic if its pre-tax foreign income (PIFO) for the years 2014–2016 is either zero or missing	Compustat
* NON_INVGRADE_RATING*	Indicator variable equal to one if firm *i* has no credit rating or a non-investment grade credit rating in the years 2014–2016, and the value of zero if firm *i* has an investment grade credit rating in these years	S&P Credit Ratings
Additional Variables for the U.S. Sample		
* CAR*	Cumulative abnormal return around the announcement of the acquisition of foreign target *i*, consistent with Hanlon et al. (2015). We calculate the return for a five-day window around the announcement date (days: *t−2* to *t* + *2*). We calculate the market return using a value-weighted market portfolio	CRSP
* LN(DEAL_VALUE)*	Natural logarithm of the deal value for target *i*	Zephyr
* DIVERSIFYING*	Indicator variable equal to one if foreign target *i* operates in a different one-digit NACE industry than the ultimate owner of the firm that acquires foreign target *i* and zero otherwise	Zephyr
* PUBLIC_TARGET*	Indicator variable equal to one if foreign target *i* is a publicly listed firm and zero if foreign target *i* is an unlisted firm	Orbis
* LN(MARKET_CAP)*	Natural logarithm of the market capitalization of target *i* in year *t−1*, as market value of equity (PRCC*CSHO)	Compustat

## Footnotes

1 A high statutory corporate income tax rate of 35%, imposed upon the repatriation of foreign income (with relief granted for foreign tax), made the tax cost of U.S. ownership of foreign assets high, distorting investment decisions by U.S. firms (Desai and Hines 2003). Moreover, the incentive to postpone the repatriation of foreign income led to substantial amounts of cash being held abroad (Foley et al. 2007; Gu 2017) and being spent, in part, on less profitable foreign acquisitions (Edwards et al. 2016; Hanlon et al. 2015; Harford et al. 2017).

2 The minimum rate is not the same for every U.S. firm due to complex U.S. expense allocation rules (Bunn 2021). In our empirical tests, we thus look at relatively low rates.

3 Lyon (2020) studies the impact of the TCJA on deal valuations. The theoretical (and expected empirical) impact of any tax reform on deal valuations is a priori less clear than for deal counts or deal probabilities, given the possibility that firms at different points along the deal value distribution may be differentially susceptible to different M&A motives. Thus, for instance, transaction volume could be increasing alongside declining valuations, if relatively small (i.e., low value) acquisitions benefit more from the tax reform than do relatively large (i.e., high value) deals. We are concerned with understanding a firm’s motives to pursue M&A, regardless of size.

4 Untaxed foreign earnings refer to the active earnings of foreign corporate affiliates that were not taxed in the United States because they were not yet repatriated. In contrast, passive earnings of foreign corporate affiliates are generally taxed by the United States on an accrual basis under Subpart F. Those earnings can be repatriated tax-free because they were taxed by the United States when earned (i.e., they are not untaxed foreign earnings).

5 Dunker et al. (2022) test for the effects of GILTI in a sample of U.S. public firms and draw similar inferences as ours from acquirer-level tests about the negative effects of GILTI on U.S. firms’ foreign acquisition incentives. In contrast, Atwood et al. (2020) conclude that GILTI incentivizes U.S. firms to acquire foreign targets. As we discuss in Section 2.1, both studies take a different approach to studying the effects of GILTI that, in our view, is more indirect than ours and relies on different data sources.

6 Dunker et al. (2022) separately examine acquisitions in low-tax countries (where the negative result is observed) and high-tax countries, which improves their ability to detect a negative effect of GILTI, relative to Atwood et al. (2020). Given their broader approach and research design, Atwood et al. (2020) cannot effectively disentangle a number of offsetting incentives of the TCJA and attribute some of their results specifically to GILTI.

7 The literature is mixed with respect to the impact of the U.S. repatriation tax on the acquisition of domestic U.S. targets by U.S. acquirers. Hanlon et al. (2015) find that repatriation taxes are positively associated with foreign but not with domestic M&A. Martin et al. (2015) find that repatriation taxes are positively associated with both foreign and domestic M&A. Harris and O’Brien (2018) find that repatriation taxes are negatively associated with domestic M&A. See Chen and Shevlin (2018) for a discussion.

8 An inversion describes the process of re-domiciling for tax purposes. Prior to the TCJA, the high U.S. statutory corporate income tax rate and the repatriation tax on foreign earnings provided an incentive for firms to move their tax domicile from the United States to a more favorable taxing jurisdiction (Babkin et al. 2017).

9 The TCJA also included a base erosion anti-abuse tax (BEAT), intended to discourage domestic firms from shifting profits out of the United States via outbound intercompany payments. We discuss BEAT in Appendix 1 but do not include it in our empirical tests. The BEAT provisions are relatively easy to avoid (Laplante et al. 2021), and we do not think they had clear incentive effects on outbound acquisitions of U.S. firms.

10 As noted, due to the complexity of U.S. expense allocation rules, the precise minimum rate that subjects foreign income to GILTI varies across firms (Bunn 2021).

11 The Biden administration has proposed to increase the GILTI rate from 10.5% to 20% in conjunction with an increase in the corporate tax rate from 21 to 28%. The Biden Administration has also proposed applying GILTI on a jurisdiction-by-jurisdiction basis to prevent U.S. firms from blending income earned in both high- and low-tax jurisdictions to avoid the GILTI tax. While these proposals were not included in the Inflation Reduction Act of 2022, if passed, they should further discourage U.S. acquisitions of profitable targets in low-tax jurisdictions.

12 Including fixed effects in nonlinear logit or probit models could cause the incidental parameters problem discussed by Allison (2009) and Greene (2004). Linear probability models are less prone to this concern and therefore are preferable in fixed-effects estimations with a binary dependent variable (Wooldridge 2010).

13 We generally winsorize continuous variables at the 1% and 99% levels.

14 We calculate *REPAT_TAX_COST*, *DOMESTIC*, and *NON_INVGRADE_RATING* over the period 2014 to 2016. We compute long-run measures to alleviate endogeneity concerns (Klassen and Laplante 2012). We choose 2016 as the end point because, with the TCJA enacted in December 2017, it is the last fiscal year entirely unaffected by the reform.

15 We calculate a firm’s repatriation tax costs in a given year consistent with Foley et al. (2007), as pre-tax foreign income multiplied by 35% less foreign income taxes. We set observations with missing values to zero and take a three-year average from 2014 to 2016.

16 *NON_INVGRADE_RATING* is negatively correlated with *LEVERAGE* (p < 0.01). Thus firms in our sample with no or a non-investment grade rating exhibit lower leverage ratios than firms with an investment grade rating, consistent with the former group having constrained access to debt markets (Faulkender and Petersen 2006).

17 We exported the data from Zephyr on December 11, 2019.

18 We can increase the sample size considerably (i.e., by approximately 35,000 observations) if, when information on the global ultimate owner is missing, we assume that the acquirer is its own global ultimate owner. We exclude these observations to be conservative in our approach and ensure that we have correctly classified the country of the acquirer’s global ultimate owner. The main results that we report later in our paper are not affected by this assumption.

19 Excluding micro deals is consistent with the approach of Bird et al. (2017). Including these observations does not affect our main results.

20 We obtain financial statement data for the years 2010 to 2018, because acquirer-level controls in Eq. (2) are lagged by one year (see Section 3.1.2).

21 Compustat uses Committee on Uniform Securities Identification Procedures (CUSIP) numbers, not ISIN. Yet each ISIN, as reported in Orbis, can be transformed into a CUSIP number by extracting the ISIN’s final six digits.

22 We focus on the completion date of a deal when defining *POST* to capture actual (i.e., completed) investment as opposed to announced investment. We find similar results when using the announcement date of a deal.

23 The results in column (6) are robust to several alternative fixed effect structures. Gaertner et al. (2020) find significant heterogeneity in stock returns of non-U.S. firms surrounding the passage of the TCJA, varying by country, industry, and firm. Thus, while the TCJA may have changed the incentives to conduct (cross-border) M&A for a certain subset of non-U.S. firms, teasing out the specific set of firms is beyond the scope of our study.

24 We split the sample at the annual median to account for the downward trend in statutory corporate income tax rates during our sample period. The mean statutory corporate income tax rate in the low-tax (high-tax) subsample equals 20.6% (30.6%), consistent with the split dividing our sample into relatively low- and relatively high-taxed foreign targets.

25 We estimate a fully interacted model to assess whether the coefficients on *POST* differ between subsamples (Allison 1999). Specifically, we interact all independent variables with an indicator variable that identifies the subsamples and re-estimate the regression on the full sample. We then conduct a one-tailed t-test to assess whether the coefficient on *POST* is smaller in columns (1), (3), and (5) than in columns (2), (4), and (6), respectively. p-values for these tests are provided in Table 3. Our results are similar when only interacting *POST* (untabulated).

26 All targets in the high-profitability and low-tax-rate subsample (column (3)) earn an annual return on tangible assets of more than 10%, consistent with the intangible income definition under GILTI. The results are similar when we split the sample based on the 10% return on tangible assets cutoff. However, we believe that splitting the sample based on median profitability is more appropriate because GILTI operates at the level of the consolidated foreign operations and discourages adding a relatively profitable target to a portfolio of foreign income.

27 Alternatively, we consider a target’s active patent holdings provided by Orbis as a proxy for high expected profits. We find (untabulated) strong evidence that the reduced likelihood of foreign targets being acquired by a U.S. firm is concentrated in the low-tax-rate and high-patent-holding subsample.

28 Like Feld et al. (2016), we control for characteristics of each potential acquirer country (*LN(GDP_CAPITA)*, *GDP_GROWTH*, *MARKET_VALUE_EQUITY*, and *EXCHANGE_RATE*) and of each potential acquirer-target country pair (*NUMBER_ACQUISITIONS*, *LN(DISTANCE)*, *NEIGHBORING*, *COMM_LANGUAGE*, *COLONY*, and *SAME_COUNTRY*). We include *MARKET_VALUE_EQUITY* and *EXCHANGE_RATE* in a second step, because data for these variables are unavailable for all potential acquirer countries, leading to a sizeable loss in sample size (see column (2) in Table 4).

29 Specifically, we assume pseudo reforms in the United Kingdom, Japan, China, Sweden, France, Canada, Germany, the Netherlands, Australia, and Switzerland.

30 Our main results are also insensitive to dropping either target or acquirer observations in countries that decreased their statutory corporate income tax rates in the post-TCJA period (untabulated).

31 Related to the results that we report in Table 5, we do consider how comparable treatment firms are to control firms in untabulated descriptive analyses. For example, firms with untaxed foreign earnings prior to the TCJA are larger and exhibit lower sales growth. To address concerns about inferences from these sample differences, we follow Gallemore et al. (2019) and apply entropy balancing to match treatment and control firms. We find similar results to those reported in Table 5 after re-estimating the acquirer-level analysis on the matched samples (untabulated).

32 Since we include firm and year fixed effects, *REPAT_TAX_COST* and *POST* are collinear with the set of firm and year indicators included in the regression and therefore subsumed in the estimation. We obtain similar results when re-estimating this and the subsequent tests without year fixed effects.

33 Additional analyses suggest that cash savings from reduced cross-border M&A may be large enough to partly finance these payout responses (untabulated). Specifically, when examining the annual dollar amount spent on foreign acquisitions, our estimate on *REPAT_TAX_COST*POST* suggests a relative reduction by 26.2%, indicating a $101.5 million decrease in the annual amount spent on cross-border acquisitions post TCJA by the average firm with untaxed foreign earnings in our sample. For comparison, Beyer et al. (2022) document annual payout increases in the range of $86 million to $206 million.

34 Our results are also robust to excluding firms (rather than setting their repatriation tax costs to zero) that report nonzero or nonmissing foreign income taxes but missing or zero pre-tax income in a given year.

35 To this end, we replace the dependent variable in Eq. (2) with the number of deals completed by firm *i* in year *t*. We re-estimate the analysis in column (1) of Table 5 using a negative binomial regression to accommodate the count-data structure of the dependent variable.

36 Pre-reform, all yearly treatment effects are insignificant (all p > 0.13). These estimates are also jointly insignificant (p = 0.82), and their sum is not significantly different from zero (p = 0.44).

37 Figure 2d again indicates that treatment and control firms exhibit similar pre-reform trends in their probability of acquiring a foreign target, since yearly treatment effects are insignificant pre-reform (all p > 0.19). Further, these estimates are jointly insignificant (p = 0.62), and their sum is not significantly different from zero (p = 0.84).

38 The potential cash-tax savings for debt-constrained firms can be substantial. Applying the statutory rate reduction of 14 percentage points and the 10.6 percentage point lower cash-tax burden on domestic operations estimated by Dyreng et al. (2020), respectively, on the post-reform domestic pre-tax income of debt-constrained firms in our sample, we obtain mean cash-tax savings ranging between $19.8 million and $26.1 million per year. For a subset of firms, these cash-tax savings are large enough to finance the cross-border acquisitions in a given year.

39 A Canadian firm could be indirectly affected by the TCJA if it plans to acquire a U.S. target. To alleviate concerns that this could affect the inferences drawn from this test and to be consistent with the target-level analysis, we limit foreign acquisitions of Canadian firms to targets located outside the United States. We obtain similar results when also excluding Canadian targets acquired by U.S. firms.

40 We again find insignificant yearly treatment effects for the pre-period (all p > 0.29). These estimates are also jointly insignificant (p = 0.67), and their sum is not significantly different from zero (p = 0.88), suggesting that U.S. and Canadian firms exhibit similar pre-reform trends in the probability to acquire a foreign target.

41 The sample for this analysis includes all foreign deals with sufficient data announced by firms in the U.S. sample. We require a minimum of five deals per target country to accommodate target-country fixed effects but note that our inferences are qualitatively unchanged without applying this restriction (untabulated).

42 The sample selection criteria follow the requirements for our global sample. As an additional step, we link deals with U.S. targets from Zephyr with financial statement data from Compustat, using the ISIN of the target (again transformed into CUSIP before merging the two datasets). As a result, the sample in this analysis is limited to publicly listed U.S. targets, following Bird et al. (2017). Since we examine public targets only, we follow Bird et al. (2017) and use market capitalization (*LN(MARKET_CAP)*) as a proxy for target size.

43 While an acquisition in some cases may trigger an acceleration of the timing of the transition tax payments on *past* foreign earnings, it does not change the overall amount the firm has to pay. Therefore the effect on the acquisition price and thus the likelihood of acquiring such targets should be low and relate primarily to the elimination of the repatriation tax on *future* foreign earnings. In most cases, the acquirer essentially steps into the shoes of the seller by filing a timely transfer agreement with the IRS with no acceleration (RSM 2018).

44 We follow Allen and Morse (2019) and use segment data to identify U.S. targets with non-U.S. sales. We recognize that non-U.S. sales do not exclusively represent export sales, which form the basis for calculating FDII (see Appendix 1). In untabulated tests, we find consistent results when using the existence of foreign operations (*DOMESTIC* = 0) to identify U.S. targets serving foreign markets.
